# Physiological cost of antibiotic resistance: Insights from a ribosome variant in bacteria

**DOI:** 10.1126/sciadv.adq5249

**Published:** 2024-11-15

**Authors:** Eun Chae Moon, Tushar Modi, Dong-yeon D. Lee, Danis Yangaliev, Jordi Garcia-Ojalvo, S. Banu Ozkan, Gürol M. Süel

**Affiliations:** ^1^ Department of Molecular Biology, School of Biological Sciences, University of California, San Diego, San Diego, CA 92093, USA.; ^2^ Department of Physics and Center for Biological Physics, Arizona State University, Tempe, AZ 85287-1504, USA.; ^3^ Sarafan ChEM-H, Stanford University, Stanford, CA 94305, USA.; ^4^ Department of Medicine and Life Sciences, Universitat Pompeu Fabra, Barcelona Research Park, Barcelona 08003, Spain.; ^5^ Center for Microbiome Innovation, University of California, San Diego, San Diego, CA 92093-0380, USA.; ^6^ Synthetic Biology Institute, University of California, San Diego, San Diego, CA 92093, USA.

## Abstract

Antibiotic-resistant ribosome variants arise spontaneously in bacterial populations; however, their impact on the overall bacterial physiology remains unclear. We studied the naturally arising antibiotic-resistant L22* ribosome variant of *Bacillus subtilis* and identified a Mg^2+^-dependent physiological cost. Coculture competition experiments show that Mg^2+^ limitation hinders the growth of the L22* variant more than the wild type (WT), even under antibiotic pressure. This growth disadvantage of L22* cells is not due to lower ribosome abundance but rather due to reduced intracellular Mg^2+^ levels. Coarse-grained elastic-network modeling of ribosome conformational dynamics suggests that L22* ribosomes associate more tightly with Mg^2+^ when compared to WT. We combined the structural modeling and experimental measurements in a steady-state model to predict cellular adenosine 5′-triphosphate (ATP) levels, which also depend on Mg^2+^. Experiments confirmed a predicted ATP drop in L22* cells under Mg^2+^ limitation, while WT cells were less affected. Intracellular competition for a finite Mg^2+^ pool can thus suppress the establishment of an antibiotic-resistant ribosome variant.

## Teaser

A resistant bacterial ribosome variant is outcompeted despite antibiotic pressure when Mg^2+^ is limited, exposing a vulnerability.

## INTRODUCTION

**Fig. 1.**
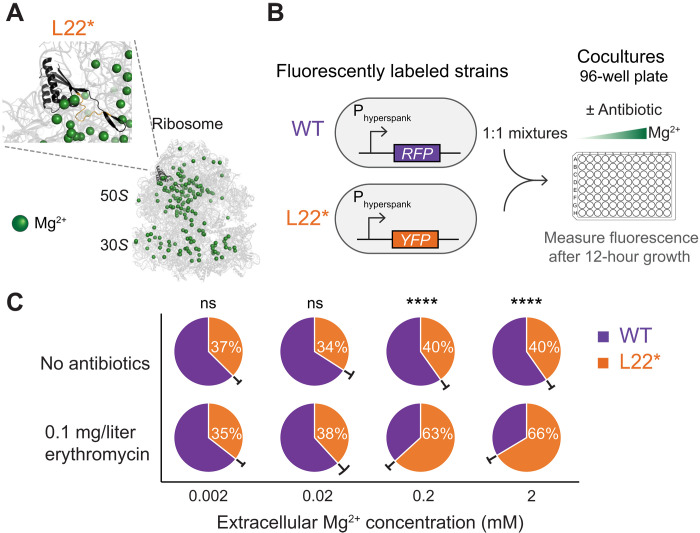
The fitness of L22* strain depends on Mg^2+^ availability. (**A**) Cartoon of the 3D ribosome structure showing the localization and magnified view of the mutated ribosomal protein L22* (black) and structural Mg^2+^ (green) of the ribosome. The duplicated region (SQINKRT duplication in 94th amino acid position) in the loop of the L22* variant is colored in orange. The ribosome structure was obtained from the Protein Data Bank (PDB: 3J9W) and is represented using PyMOL (The PyMOL Molecular Graphics System, Version 3.0 Schrödinger, LLC; https://pymol.org/). The 3D structure of the L22* subunit was obtained using AlphaFold (*50*, *51*). (**B**) Schematic of coculture competition assay. WT and L22* strains were labeled with distinct fluorescent proteins. The cocultures were started with a 1:1 mixture of strains and measured after 12 hours of growth in a 96-well plate. As indicated, conditions tested varied in terms of extracellular Mg^2+^ concentrations and the presence/absence of an antibiotic [erythromycin (0.1 mg/liter)]. (**C**) Pie charts summarizing the percent L22* in cocultures at the end of 12-hour growth with error bars represented outside the pie chart between the two slices in black lines (mean ± 95% confidence interval; *n* ≥ 5 experiments per condition). Using a between-subjects two-way analysis of variance (ANOVA), main effects of antibiotic presence [*F*(1,40) = 155.7, *P* < 0.0001], Mg^2+^ [*F*(3,40) = 82.39, *P* < 0.0001], and interaction [*F*(3,40) = 45.09, *P* < 0.0001] were significant. Using post hoc Bonferroni multiple comparison test, ns = not significant, ****P < 0.0001 between antibiotic presence at each extracellular Mg^2+^ condition.

Ribosomes are a major target for naturally occurring antibiotics (*1*). Studies have identified the spontaneous emergence of ribosome variants in bacteria that confer resistance to naturally occurring antibiotics (*2*–*6*). In *Bacillus subtilis*, for instance, the spontaneously arising ribosome variant L22* has a sequence extension of the short loop comprising its L22 subunit (Fig. 1A) (*7*). This L22* mutation provides resistance to erythromycin (*7*), which binds near the mutation site. In addition, L22* also provides relatively moderate resistance to spectinomycin (*8*), which binds in the 30*S* subunit, distant from the L22 subunit. Given that ribosome variants such as L22* have not replaced the wild-type (WT) ribosome, one can assume that resistance-conferring ribosome variants carry a physiological cost in the absence of antibiotic selection pressure (*9*, *10*). However, it has been difficult so far to elucidate the specific physiological costs associated with such ribosome variants (*11*, *12*). Elucidating the physiological cost-benefit balance in bacterial strains with mutant ribosome variants could reveal alternative avenues to combat the antibiotic crisis.

Here, we focused on magnesium ion (Mg^2+^) availability because ribosomes require magnesium ions for their stability and functionality, through neutralizing electrostatic repulsion and spatially coordinating ribosomal RNA (rRNA) functional groups (*13*–*15*). High-resolution crystal structures of bacterial ribosomes have revealed that each complex contains more than 170 structural Mg^2+^ (*16*), which are an integral part of the ribosome complex (Fig. 1A) (*17*). Undoubtedly, many more Mg^2+^ associate transiently with ribosomes (*18*). Other inorganic cations such as potassium also play a role in ribosome stability and function (*19*), but these cannot fully substitute for the structural stability and activity of the ribosome provided by Mg^2+^ (*20*, *21*). What makes Mg^2+^ unique is that it has a relatively small atomic number (*Z* = 12) and a divalent positive charge (*q* = +2*e*). The ribosome complex assembly strongly depends on Mg^2+^ concentration (*22*–*26*). In addition, Mg^2+^ has been shown to help bacteria cope with ribosome-targeting antibiotics (*8*). Therefore, a more comprehensive understanding of ribosomes is more likely to emerge by taking into account their interactions with this metal cation.

It is important to note that Mg^2+^ is also an essential cofactor for other cellular processes and, in particular, serves as the counterion for adenosine 5′-triphosphate (ATP), the energy currency of cells. Mg^2+^-bound ATP is the major biologically active form of cellular ATP (*27*–*29*). Together with ribosomes, ATP thus constitutes the largest intracellular store of chelated Mg^2+^ (*30*). Studies have also reported reduced ATP and ribosome production in response to Mg^2+^ deficiency in other organisms (*28*–*32*). In line with these previous insights into the broader functional role of Mg^2+^, here, we identify a specific physiological cost associated with the antibiotic-resistant L22* ribosome variant, which arises from a zero-sum competition between ribosomes and ATP for a shared and limited Mg^2+^ pool. The benefit of antibiotic resistance thus becomes pitted against the need for robustness against extracellular Mg^2+^ limitations, which can favor the WT ribosome variant.

## RESULTS

### The fitness of L22* strain depends on Mg^2+^ availability

To directly determine the fitness differences between the WT and L22* strains, we cocultured the two strains under varying Mg^2+^ concentrations both in the presence and absence of antibiotics (Fig. 1B). We used a sublethal concentration (0.1 mg/liter) of erythromycin antibiotic to allow growth of the two strains. We tracked each strain in the coculture over time using chromosomally integrated transcriptional reporters expressing spectrally distinct fluorescent proteins (Fig. 1B). After 12 hours of growth, we find that the coculture behavior does not depend only on antibiotic presence alone but also on magnesium availability (Fig. 1C). In the absence of antibiotic pressure, both strains coexisted with similar fractions. However, in the presence of antibiotic pressure, we observed a tipping point between 0.02 and 0.2 mM Mg^2+^ at which the dominant strain switches. Specifically, at Mg^2+^ concentrations above 0.2 mM, the resistant L22* strain dominated, as would be expected given the presence of antibiotics. Unexpectedly, at low Mg^2+^ concentrations (<0.02 mM), the WT strain dominated over the L22* despite the presence of an antibiotic (Fig. 1C). This result shows that direct competition between WT and L22* not only is not exclusively determined by antibiotic pressure but also depends on Mg^2+^ availability. These data demonstrate that the fitness of the antibiotic-resistant L22* strain is more complex, necessitating consideration of extracellular Mg^2+^ availability.

### The L22* ribosome reduces the free Mg^2+^ pool in the cell

**Fig. 2.**
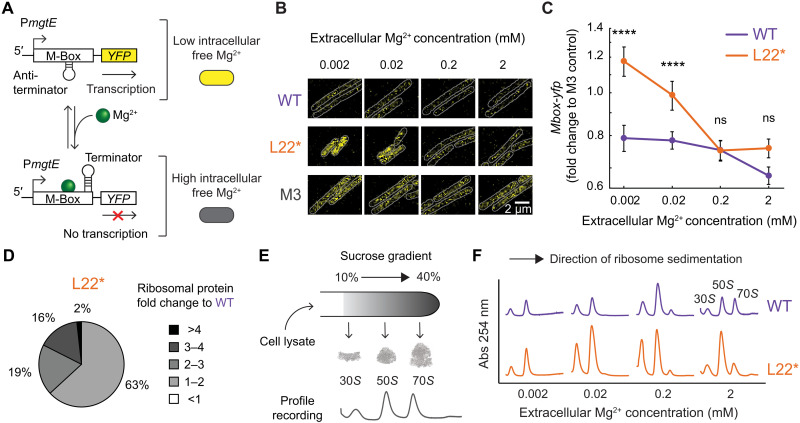
The L22* mutation reduces the free Mg^2+^ pool in a cell and increases the ribosome content of the cell. (**A**) Schematic of an M-box riboswitch-based reporter used in this study. A cell will turn on a *yfp* gene when it experiences low intracellular free Mg^2+^ concentrations. (**B**) Representative snapshots of YFP expression at different extracellular Mg^2+^ concentrations. Each cell is outlined in gray based on the corresponding phase image. WT and L22* strains have the same riboswitch-reporter Mbox-*yfp* expressed, and the M3 strain has Mbox^M3^-*yfp* (M-box mutant that expresses YFP independent of the intracellular Mg^2+^ level) expressed in WT. Scale bar, 2 μm. (**C**) Mbox-*yfp* signals in WT and L22* strains relative to Mbox^M3^-*yfp* control are plotted for different extracellular Mg^2+^ concentrations (mean ± 95% confidence interval; *n* ≥ 4 images from three experiments; *n* ≥ 10 cells analyzed from each image). Using a between-subjects two-way ANOVA, main effects of strain [*F*(1,41) = 65.23, *P* < 0.0001], Mg^2+^ [*F*(3,41) = 39.30, *P* < 0.0001], and interaction [*F*(3,41) = 15.81, *P* < 0.0001] were significant. Using post hoc Bonferroni multiple comparison test, ns = not significant, ****P < 0.0001 between strains at each extracellular Mg^2+^ condition. (**D**) Pie chart showing fold change to WT levels of ribosomal proteins in the L22* strain at 2 mM extracellular Mg^2+^ concentration (*n* = 2 samples for WT; *n* = 1 sample for L22*). Using a repeated measures two-way ANOVA, main effects of strain [*F*(1,1) = 629.8, *P* = 0.0254], protein [*F*(1,1) = 1293, *P* = 0.0177], and interaction [*F*(53,53) = 30.64, *P* < 0.0001] were significant. (**E**) Schematic of a sucrose gradient ribosome sedimentation profile used in the study. 30*S*, 50*S*, and 70*S* peaks are labeled in the example profile. (**F**) Ribosome sedimentation profiles of the WT and L22* strains at various extracellular Mg^2+^ concentrations.

Given the observed sensitivity of the L22* strain to low Mg^2+^ conditions, we investigated the intracellular free Mg^2+^ in live bacterial cells from both strains. To that end, we generated a genetic fluorescent reporter using a previously characterized *B. subtilis* native M-box riboswitch that is sensitive to free Mg^2+^ levels (Fig. 2A) (*33*). The M-box riboswitch is part of the 5′ untranslated region (5′UTR) of the *mgtE* transcript, which codes for a Mg^2+^ importer, and its function is to reduce *mgtE* expression upon binding of free Mg^2+^. In this way, the M-box riboswitch enables cells to express the Mg^2+^ transporter MgtE only when needed. By fusing the 5′UTR of *mgtE* with a *yfp* gene, we constructed a live-cell imaging reporter that indicates a shortage of free Mg^2+^ in cells. We used the activity of the reporter to compare the levels of free Mg^2+^in the WT and L22* strains at different extracellular Mg^2+^ concentrations. Bacteria were grown in minimal defined media (MSgg) and then imaged at single-cell resolution on carrageenan agar pads (Fig. 2B). As a control, we used the M-box with a previously characterized M3 mutation in the aptamer domain that is known to make the riboswitch active independently of the Mg^2+^ levels by disabling their binding (*33*). As expected, yellow fluorescent protein (YFP) expression in the M3 control remained high, regardless of extracellular Mg^2+^ concentrations (Fig. 2B). We quantified the fluorescence signal and normalized the results from WT and L22* strains to the M3 control strain to account for potential experimental variability. Results show that the L22* strain is deficient (the reporter signal is high) in maintaining free intracellular Mg^2+^ at low extracellular Mg^2+^ levels, when compared to WT. Notably, the concentration range of extracellular Mg^2+^ over which we observe a drop in free Mg^2+^ level (increase in the YFP signal) corresponds to the region where we also observe a switch in dominant strain in the coculture in the presence of antibiotics (compare Fig. 1C and Fig. 2C). We also find a statistically significant increase in the MgtE protein expression in the L22* mutant from a proteomic analysis (fig. S1A), confirming the results of the riboswitch reporter assay. In other words, the WT strain is robust and able to maintain free Mg^2+^ levels despite low extracellular Mg^2+^ concentrations, while the L22* strain exhibits quantifiable sensitivity to extracellular Mg^2+^ limitation.

### The L22* mutation increases the ribosome content in the cell

Given that ribosome content determines growth rate in bacteria, we asked if the observed growth deficiency of the L22* strain was due to low ribosome abundance. We used mass spectrometry (MS) proteomics to perform a comparative analysis of the ribosomal protein levels in the L22* strain relative to the WT strain at 2 mM extracellular Mg^2+^ concentration. The results revealed broad up-regulation of ribosomal proteins in the L22* strain (Fig. 2D). The up-regulation of ribosomal proteins is specific as total protein expression is not increased in the L22* when compared to the WT (fig. S1B). Next, we analyzed the assembly state of the ribosomes by measuring their sedimentation profile using a sucrose gradient centrifugation of cell extracts. The absorbance profiles obtained after centrifugation indicate ribosome subunits sedimenting through the sucrose gradient at different rates (Fig. 2E). The results show that, for all extracellular Mg^2+^ concentrations tested, the L22* mutant had a greater content of ribosomal subunits compared to WT (Fig. 2F). These findings show that the inability of L22* to grow effectively at low Mg^2+^ is not due to lower ribosome abundance.

### The L22* mutation increases Mg^2+^-ribosome association

**Fig. 3.**
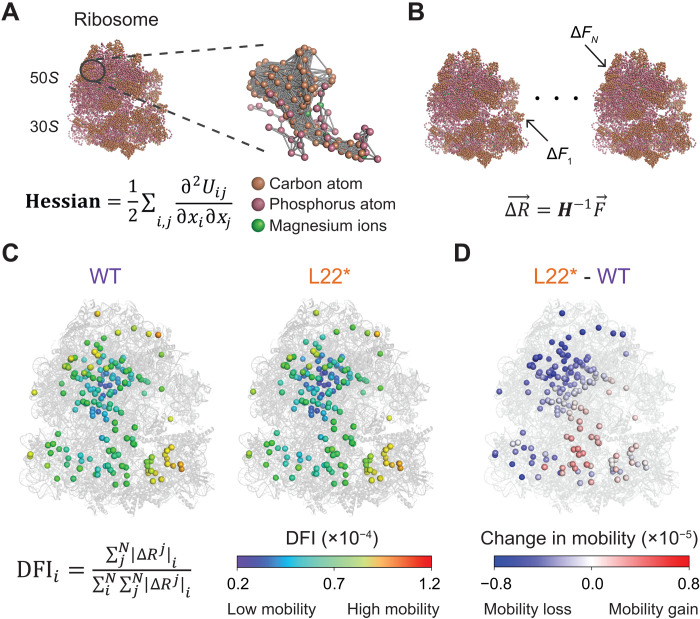
An ENM reveals the dynamic flexibility of ribosomal components. (**A**) Cartoon representation of a ribosome in a coarse-grained ENM. Each colored ball denotes each node as described in the panel, and each gray line accounts for an interaction between the nodes. Hessian (*H*) is defined as presented in the equation at the bottom. The ribosome structure was obtained from the Protein Data Bank (PDB: 3J9W) and is represented using PyMOL. (**B**) Cartoon illustration of sequential perturbative forces applied to each node. (**C**) Cartoon illustrating the DFI of Mg^2+^ in WT and L22* ribosomes. The equation on the left defines the DFI score of a node. A shared color bar among WT and L22* ribosomes is at the bottom. The color scale is normalized to the maximum and minimum DFI values. The 3D structure of the L22 subunit in the L22* variant was obtained using AlphaFold. (**D**) Ribosome cartoon of change in Mg^2+^ mobility as the differences of DFI of Mg^2+^ in the L22* ribosome from those in the WT ribosome.

Given the higher L22* ribosome content in the cells, we asked if the L22* ribosomes exhibited a different affinity for the structural Mg^2+^ ions present in the ribosomal crystal structure. Because this question is difficult to measure experimentally, we turned to a computational framework. Specifically, we used a previously described coarse-grained elastic network model (ENM) where the dynamics of any molecular complex are dictated by a network of intra- and intermolecular interactions among domains and subunits and the intrinsic thermal fluctuations of atomic positions (*34*). An ENM has proven effective in elucidating and predicting the biologically relevant intramolecular interactions that underlie the structure-encoded dynamics of biomolecules and have been used in the past to successfully study interdomain motions in the ribosome (*35*–*38*). In this mechanical model, atoms are modeled as nodes and interactions as springs connecting them (*39*–*41*). We accounted for the interactions between all the alpha carbon atoms from the protein chains, the phosphorus atoms from the RNA, and the structural Mg^2+^ (Fig. 3A). We then applied a mechanical force (Brownian kick) to each node and calculated the average response of the entire network, averaged over multiple isotropic force directions (Fig. 3B). Thereby, we sequentially perturbed each node of the ribosome complex, one at a time, and then computed the response displacement Δ*R* for each node according to linear response theory. From this approach, we obtained the dynamic flexibility index (DFI) (*42*–*45*) for each node, which is the net displacement of a given position relative to the value when every node is individually perturbed (Supplementary Text) (Fig. 3C). Therefore, the DFI score of a node represents its relative mobility upon physical perturbations (such as intermolecular forces and stochastic thermal noise). Nodes with a lower DFI score have lower mobility, suggesting a higher association with the network. On the other hand, nodes with a higher DFI score would be more susceptible to fluctuations, suggesting a lower association and higher mobility. The change in DFI profiles upon mutations is highly correlated with the binding affinity (*46*–*49*). Here, DFI analysis revealed that the Mg^2+^ near the center of the ribosome have lower mobility, compared with those toward the edge (Fig. 3C, left). Consequently, DFI unveils the relative association strength of Mg^2+^ with the rest of the ribosome, making it possible to map this model onto the ribosome structure.

Using the DFI maps, we examined if the ribosomal association with Mg^2+^ differs between WT and L22* ribosomes. Our model examines ribosome crystal structures with a fixed number of structural Mg^2+^ ions and does not predict the addition of new Mg^2+^ binding sites in the L22* mutant ribosome. Instead, it assesses the changes in the flexibility of existing Mg^2+^ ions and their affinity to the ribosome. Specifically, we obtained the three-dimensional (3D) structure of the L22 subunit in the L22* variant using AlphaFold (Fig. 1A) (*50*, *51*). DFI analysis of the L22* ribosome reveals changes in the DFI values for all the structural Mg^2+^ (Fig. 3C, right). To determine how the L22* ribosome variant affects its interaction with Mg^2+^, we subtracted the DFI of Mg^2+^ in the WT ribosome from those in the L22* ribosome. We found a net loss in the mobility of the Mg^2+^ in the mutant strain (Fig. 3D). Our findings indicate that the L22* mutation affects Mg^2+^ binding at distal sites, similar to allosteric regulation. Specifically, the mutation reduces Mg^2+^ mobility, suggesting increased binding affinity and a higher probability of Mg^2+^ association at these locations. As a control, when the same DFI analysis was done on carbon and phosphorus atoms, the change in the fraction of those nodes was much smaller (fig. S2, A to C). This model suggests that the L22* mutation has a higher effect on the dynamics of Mg^2+^, compared to the rest of the ribosomal components. In other words, the model shows that there is an overall increase in the association of Mg^2+^ in the L22* ribosome. These results were consistent with other ribosome structures (fig. S3) and with all atomistic molecular dynamics (MD) simulations (fig. S4). In contrast, DFI analysis using the ENM of another ribosome variant, which carries a deletion of the L34 ribosomal subunit (ΔL34) (*8*, *52*), shows very little to no change in the Mg^2+^ association to the ribosome (fig. S5). Together, these models suggest that the L22* mutation specifically increases the association of Mg^2+^ with the ribosome.

### The L22* ribosome reduces ATP availability in the cell

**Fig. 4.**
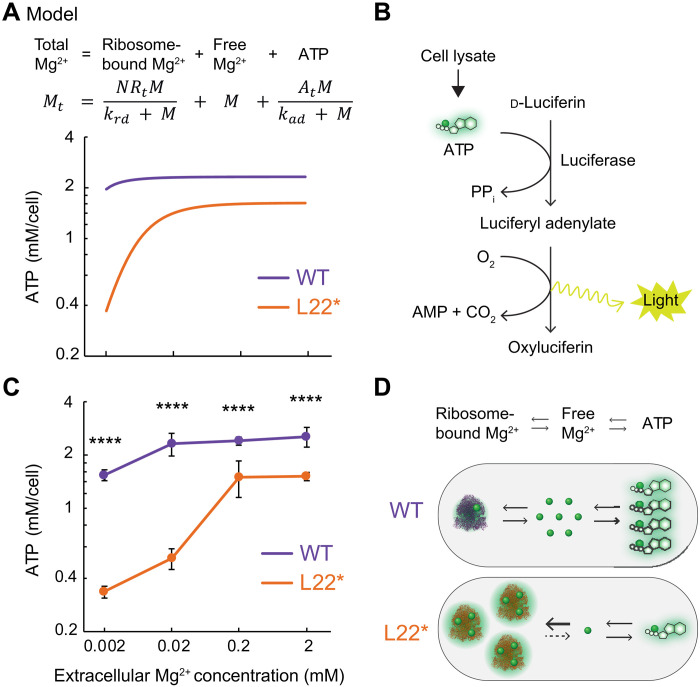
The L22* ribosome reduces ATP availability. (**A**) Steady-state model and prediction results of active ATP levels for varying extracellular Mg^2+^ concentrations in WT and L22* strains. Shared *x* axis labels are indicated in (C). (**B**) Schematic of a bioluminescence-based assay (Roche Scientific, 11-699-709-001) used in this study to determine the ATP level. PP_i_, inorganic pyrophosphate; AMP, adenosine 5′-monophosphate. (**C**) Intracellular ATP concentrations of each strain at different extracellular Mg^2+^ concentrations (mean ± 95% confidence interval; *n* = 5 experiments; *n* ≥ 2 samples per experiment). The absorbance values were converted using the standard curve shown in fig. S7A. Using a between-subjects two-way ANOVA, main effects of strain [*F*(1,32) = 621.5, *P* < 0.0001], Mg^2+^ [*F*(3,32) = 105.7, *P* < 0.0001], and interaction [*F*(3,32) = 16.29, *P* < 0.0001] were significant. Using post hoc Bonferroni multiple comparison test, ****P < 0.0001 between strains at each extracellular Mg^2+^ condition. (**D**) Cartoon description of the summary finding that the L22* ribosome reduces the availability of free Mg^2+^ and ATP.

Using the DFI modeling results together with the experimentally measured free Mg^2+^ and ribosome content data described above, we constructed a simple mathematical steady-state model of intracellular Mg^2+^ (Supplementary Text). The model considers three potential states in which Mg^2+^ can be present within a cell: free, bound to ribosomes, and bound to ATPs. Informed by our data, the L22* strain is modeled to have a higher abundance of ribosomes and increased affinity to Mg^2+^. Consequently, the model predicts a large decrease in active ATP upon reducing extracellular Mg^2+^ in the L22* strain, when compared with WT (Fig. 4A). This behavior is robust over a large range of parameter values (fig. S6A). The model demonstrates that, at least for a simplified system considering three main potential pools of Mg^2+^, changes in the ribosome abundance and their Mg^2+^ affinity affects the level of Mg^2+^ bound ATP in the cell.

To experimentally test our modeling prediction that the L22* strain would have reduced active ATP levels, we used a bioluminescence-based assay to quantify ATP in the L22* and WT strains. The assay uses the ATP-Mg^2+^ dependency of luciferase, which catalyzes the oxidation of luciferin along with the emission of green light (*53*). The luciferase reagent used contains 10 mM Mg^2+^, not including any Mg^2+^ present in the cell lysate. Given that the intracellular ATP level in bacterial cells is 1 to 5 mM (*54*–*57*), the high concentration of Mg^2+^ in the reagent ensures that any ATP present in the lysate, whether initially bound or unbound to Mg^2+^, will be effectively assayed. In short, the assay detects total ATP in cells. Cell lysates containing ATP were exposed to the reaction, and the resulting light emission was quantitatively measured (Fig. 4B and fig. S6A). In agreement with our predictions, we find that the L22* strain has a lower active ATP concentration when extracellular Mg^2+^ availability is low (Fig. 4C). Specifically, ATP levels drop in the L22* strain when extracellular Mg^2+^ concentrations are reduced from 0.2 to 0.02 mM. This sensitivity of ATP levels in the L22* strain to a reduction in extracellular Mg^2+^ is again consistent with the switch in dominant strain in coculture and the drop in the free Mg^2+^ (compare Fig. 1C, Fig. 2C, and Fig. 4C). In other words, we observe that both intracellular free Mg^2+^ and ATP levels are low when cells with L22* ribosomes experience extracellular Mg^2+^ limitation. In contrast, the ΔL34 ribosome variant strain (which was predicted to have a negligible change in its Mg^2+^ association compared to the WT ribosome, as mentioned above) does not exhibit such a drop in ATP levels upon reduced extracellular Mg^2+^ concentrations (fig. S7B). From these multiple lines of evidence, we conclude that increased sequestration of Mg^2+^ by the L22* ribosomes constitutes a physiological cost to the L22* strain, by increasing its dependence on extracellular Mg^2+^ availability (Fig. 4D). Concurrently, our results also show that the WT ribosome allows cells to maintain a relatively stable level of both free Mg^2+^ and active ATP across a broad range of extracellular Mg^2+^ concentrations.

## DISCUSSION

Together, our results suggest that the benefit of antibiotic resistance provided by the L22* ribosome could be pitted against the environmental robustness of the WT ribosome under limited extracellular Mg^2+^ concentrations. Consequently, the L22* ribosome variant could be favored or suppressed depending on which of the two environmental factors (antibiotic pressure or Mg^2+^ limitation) exerts a stronger selection pressure. In other words, the need for bacteria to cope with variable environmental conditions (extracellular Mg^2+^ availability) could outweigh the advantage provided by an antibiotic-resistant ribosome variant. Specifically, the physiological cost associated with the L22* variant appears to stem from Mg^2+^ sequestration by the ribosome that depletes the intracellular free Mg^2+^ pool, which, in turn, limits Mg^2+^ availability for ATP.

Our study uses the DFI model to computationally predict how the L22* mutation affects Mg^2+^ ions that are part of the ribosome crystal structure. Modeling predicts a net loss in the mobility of Mg^2+^ in the L22* mutant compared to the WT, indicating a higher association with Mg^2+^. This increased association of Mg^2+^ with the ribosome in the L22* mutant would then, in turn, reduce the pool of free intracellular Mg^2+^, especially under low extracellular Mg^2+^ conditions. Tighter association of the L22* ribosome variants and its increased abundance would thus act as a sink for intracellular Mg^2+^, thereby limiting its availability for cellular ATP.

While ATP is a vital energy source, ribosome abundance is known to determine the growth and overall cellular fitness of bacteria (*58*, *59*). We observed that, under low Mg^2+^ conditions, WT exhibits not only higher ATP levels (Fig. 4C) but also lower ribosome abundance compared to L22* (Fig. 2F). This indicates the interplay by which these two metrics (ATP and ribosomes) can influence the bacterial fitness. In the absence of antibiotics, the lower ribosome abundance in the WT strain may offset the advantages of its elevated ATP levels, leading to a relatively similar fitness between the WT and L22 strains across the Mg^2+^ concentration gradient, as shown in Fig. 1C. Our results further show that a sublethal dose of antibiotics can expose the coupling between ATP and ribosomes through their shared dependence on Mg^2+^ and its impact on bacterial fitness.

Our work highlights the importance of considering the interaction of Mg^2+^ with ribosomes. Decades of research on ribosomes have typically focused on the protein subunits and RNA components, and far less is known about their interactions with Mg^2+^. Here, we integrated atomic-scale mathematical models with experimental measurements of ion concentrations and single-cell resolution physiological metrics to gain insight into the broader functional role of Mg^2+^. Our study shows that Mg^2+^ ions are not merely counterions for negatively charged phosphate groups and amino acids but can also function to couple two fundamental components of living cells, namely, ribosomes and ATP. We postulate that Mg^2+^ can provide intracellular information about the state of ribosomes. Our work thus points to the possibility that other metal ions, also shared between different molecules and processes, could similarly function as coordinating signals, hinting at additional functional roles for the many inorganic metal ions that are present in all living cells (*60*). Furthermore, we note that inorganic metals supplement the function of many organic molecules. Mg^2+^ binding to such proteins can be rapid and reversible, which could lead to mismetallation and potential disruption of Mg^2+^-dependent protein functions (*61**,* *62*). However, Mg^2+^ ions that can be identified in crystal structures are by definition more stably associated and thus these ribosome sites are less prone to the risk of mismetallation.

Our modeling predicts that the imbalanced Mg^2+^ “tug-of-war” between ribosomes and ATP applies for the L22* but not the ΔL34 ribosome variant. This is an unexpected finding because ΔL34 ribosomes have been previously described to be defective in the formation of 70*S* ribosomes, and this deficiency was shown to be rescued by Mg^2+^ supplementation (*52*). Despite their need for structurally stabilizing Mg^2+^, the ΔL34 ribosomes do not seem to be able to imbalance the Mg^2+^ competition and thus do not cause a drop in ATP levels, which stands in contrast to the L22* variant. This experimentally observed difference between ΔL34 and L22* ribosomes is consistent with our ENM modeling results, which predict a higher association of L22* with Mg^2+^ when compared to ΔL34 ribosomes. According to our modeling, local changes in a network of interactions due to mutations within the ribosome can even result in effects at a distance, similar to the well-known concept of allosteric regulation that defines the function of many proteins (*18*).

As our comparison of L22* and ΔL34 ribosomes shows, not all ribosome variants will be subject to the zero-sum Mg^2+^ competition postulated here. It will be interesting to pursue what other mechanisms may be in play to prevent other spontaneously emerging ribosome variants from replacing the WT form. Despite the fact that an imbalanced tug-of-war may not impose a physiological cost to every ribosome variant, it is noteworthy that all ribosomes do depend on stabilizing Mg^2+^ for their stability and function. We note that the Mg^2+^ concentration in human serum is ~1 mM (*63*). This relatively high Mg^2+^ concentration could perhaps explain why blood-borne infections are so prevalent and difficult to treat. In addition, Mg^2+^ also plays a crucial role in the bacterial cell wall, whose biosynthesis is inhibited by the major class of beta-lactam antibiotics. Elucidating possible Mg^2+^-dependent mechanisms in beta-lactam–resistant mutants might thus be helpful to deepen our understanding of drug resistance and bacterial fitness. We hope that our work can help identify conditions that hinder antibiotic-resistant strains without requiring development of new antibiotics. It may be possible to identify approaches to limit the prevalence or persistence of bacterial strains with antibiotic resistance by targeting intracellular Mg^2+^ levels. Such alternative avenues of control are worth pursuing, given that spontaneously arising antibiotic-resistant bacteria contribute to the public health threat posed by the antibiotic crisis.

## MATERIALS AND METHODS

### Bacterial strains

The *B. subtilis* strains used in this study are listed in table S1. Briefly, L22* is an *rplV* variant bearing a seven–amino acid (SQINKRT) duplication in the 94th position, which is a spontaneously arising mutation that predominantly provides resistance against erythromycin (*7*). ΔL34 is a deletion of rpmH with chloramphenicol resistance cassette for selection (Cm^R^). The full-length *mgtE* 5′UTR construct containing M-box was adapted from (*33*), and we denote it Mbox in strain names for simplicity. *SacA*::Mbox*-yfp* strain is generated using the ECE174-based integration vector (ECE174-Mbox*-yfp*) to insert Mbox*-yfp* into the *sacA* locus. It was constructed by Gibson assembly of two polymerase chain reaction (PCR) products. The ECE174-*yfp* (Cm^R^) vector was PCR amplified using the primers GS450 and GS351. The full-length *mgtE* 5′UTR was PCR amplified using the primers GS2462 and GS2463. The overlapping ends that facilitate Gibson assembly are indicated with lowercase letters. The M3 mutation in the M-box riboswitch (Mbox*^M3^*) was created using the primers GS2465 and GS2472 (mutation site indicated with lowercase letters). The DNA sequences of the primers are listed in table S2. *AmyE*::P_hyperspank_-*yfp* was constructed by replacement of *mKate2* with *yfp* in *amyE*::P_hypserpank_-*mKate2* and was used for transformation in the L22* background strain. All transformed strains were confirmed by sequencing.

### Growth conditions

Desired *B. subtilis* strains were streaked on a fresh LB agar plate [with chloramphenicol (5 mg/liter) or erythromycin (5 mg/liter) when appropriate] a day before the experiment and incubated at 37°C overnight. A few colonies of the desired strain were used for inoculation in LB or MSgg media [5 mM potassium phosphate (pH 7.0), 100 mM Mops (pH 7.0), 700 μM CaCl_2_, 50 μM MnCl_2_, 100 μM FeCl_3_, 1 μM ZnCl_2_, 2 μM thiamine, 0.5% glycerol, and 0.5% glutamate] with indicated final MgCl_2_ concentrations and grown at 37°C with shaking. MSgg media were made from stock solutions immediately before experiments, and the stock solution of glutamate was made fresh every 2 days.

For coculture competition assays, riboswitch reporter assay, and ATP measurements, a few colonies were inoculated into 2 ml of the MSgg medium with indicated MgCl_2_ concentrations and grown for 4.5 hours prior to testing.

For proteomics samples, colonies were grown in 1 ml of LB for 3.5 hours. Cultures were washed with MSgg media of indicated MgCl_2_ concentrations, diluted 100 times, and further incubated in MSgg at 37°C with shaking for 16 hours. A total of 50 optical density at 600 nm (OD_600_) units of cells were collected per sample. Cultures were pelleted at 4000 rpm for 10 min.

For ribosome sedimentation profiles, colonies were inoculated in 50 ml of LB culture and grown at 37°C with shaking overnight. Cultures were washed and used to inoculate 200 ml of MSgg media of indicated MgCl_2_ concentrations at an initial OD_600_ of 0.04. The cultures were grown to an OD_600_ of 0.8, pelleted at 4696*g* for 5 min, resuspended in 500 μl of a lysis buffer [1X B-Per (Thermo Fisher Scientific, 78243), 1X Halt protease inhibitor cocktail EDTA-free (Thermo Fisher Scientific, 87785), 5.5 mM 2-mercaptoethanol, DNase I (5 U/ml), and RNasin Ribonuclease Inhibitor (8 U/ml; Promega, N2511)], and incubated at room temperature for 15 min. The lysates were clarified by spinning at 15,000*g* for 5 min.

### Coculture competition assay

Two hundred microliters of 0.02 OD_600_ culture was used for each sample in a clear-bottom, black 96-well plate sealed with lid. For the competition experiment, the two strains were mixed in a 1:1 ratio. Isopropyl-β-d-thiogalactopyranoside (1 mM) and a final concentration (0.1 mg/liter) of erythromycin were used when appropriate. Plates were incubated in an Infinite 200 PRO plate reader (Tecan) at 30°C with shaking over a 12-hour period, with 15-min interval reads. OD_600_ readings were taken with pathlength correction. mKate2 fluorescence was excited at 588 nm, and emission was measured at 633 nm with a gain of 170. YFP fluorescence was excited at 515 nm, and emission was measured at 545 nm with a gain of 120. Fluorescence readings were normalized by the maximum intensity reached by a monoculture, which occurs at the end of 12-hour incubation under a 2 mM extracellular Mg^2+^ condition without erythromycin. Percent L22* in coculture was calculated as mKate2 fluorescence divided by the sum of YFP and mKate2 fluorescence. Fluorescence readings were normalized so that timepoint 0 starts at 50% to reflect the 1:1 mixed ratio at the start of the coculture.

### Time-lapse microscopy

The riboswitch reporter of *B. subtilis* cells was monitored with time-lapse fluorescence microscopy. The OD_600_ of cultures were adjusted to 0.02 with the same growth media and was applied onto a 1.5% κ-carrageenan (Sigma-Aldrich, 11114-20-8) pad made with the MSgg medium with desired final MgCl_2_ concentrations. The pads were covered, left to air dry for 1 hour at 30°C, and placed into a coverslip-bottom WillCo dish for imaging. We recorded phase-contrast and YFP fluorescence images at 30°C using an Olympus confocal laser scanning microscope FV3000 with a motorized stage (ASI). Single layers of cells were imaged every 20 min under a 60X objective lens with 1.5X zoom. Collected images were processed with ImageJ (National Institutes of Health, http://imagej.net/nih-image/).

### Riboswitch reporter assay for free Mg^2+^ measurement

Trainable Weka segmentation plugin from FIJI [ImageJ (*64*)] was used to segment cells from each phase-contrast image. Multilayer of cells, spores, and background noise were eliminated through size filtering and screening. A mask created for each image was obtained as regions of interest and applied to the corresponding YFP images to extract fluorescence intensity for each segmented cell. The fluorescence intensity of cells was averaged per image. Then, the mean fluorescence intensity under a certain condition was calculated as the average of all images for the condition. Fold change relative to M3 control (*SacA*::Mbox*^M3^-yfp* in WT background) was calculated by dividing by the mean fluorescence intensity of M3 control at a given extracellular Mg^2+^ concentration.

### MS proteomics

Sample preparation, data acquisition, and data analysis for MS proteomics were adapted from (*8*).

#### Sample preparation

Cell pellets were suspended in 200 μl of a cold extraction buffer [6 M guanidine hydrochloride (GdnHCl)/20 mM Triethylammonium bicarbonate buffer (TEAB)/10 mM tris(2-carboxyethyl)phosphine (pH 7)]. One hundred microliters of 0.5-mm ZrO_2_ beads was added. Cells were lysed by shaking in a Bullet Blender tissue homogenizer (Next Advance Inc.) at speed 8 for 3 min. Proteins were denatured by heating at 75°C for 10 min. Samples were diluted six times to 1 M GdnHCl with 20 mM TEAB. Proteins were first digested with Lys-C (Wako Chemicals, 125-05061) at 37°C for 15 min. The protein solution was further diluted two times to 0.5 M GdnHCl with 20 mM TEAB and digested with trypsin (Roche, 03 708 969 001) overnight. Digested peptides are purified on Waters Sep-Pak C18 cartridges, eluted with 60% acetonitrile. TMTpro labeling is performed in 50% acetonitrile/20 mM TEAB (pH 7). Tandem mass tag (TMT) labeling efficiency is checked by liquid chromatography–tandem mass spectrometry (LC-MS/MS) to be greater than 99%. Labeled peptides from different samples are polled together for 2D-nanoLC-MS/MS analysis. An Agilent 1200 high-performance LC system is used to deliver a flow rate of 600 nl min^−1^ to a custom three-phase capillary chromatography column through a splitter. Column phases are a 30-cm-long reversed-phase (RP1, 5-μm Zorbax SB-C18, Agilent), 8-cm-long strong cation exchange (SCX, 3-μm PolySulfoethyl, PolyLC), and 40-cm-long reversed-phase 2 (RP2, 3.5-μm BEH C18, Waters), with the electrospray tip of the fused silica tubing pulled to a sharp tip (inner diameter < 1 μm). Peptide mixtures are loaded onto RP1, and the three sections are joined and mounted on a custom electrospray adapter for on-line nested elutions. Peptides are eluted from the RP1 section to SCX section using a 0 to 70% acetonitrile gradient for 60 min and then are fractionated by the SCX column section using a series of 15-step salt gradients of ammonium acetate over 20 min, followed by high-resolution reversed-phase separation on the RP2 section of the column using an acetonitrile gradient of 0 to 70% for 150 min.

#### Data acquisition

Spectra are acquired on a Q Exactive mass spectrometer (Thermo Fisher Scientific) operated in positive ion mode with a source temperature of 325°C and spray voltage of 2.5 kV. Automated data-dependent acquisition was used on the top 15 ions with an isolation window of 1.0 Da and collision energy of 30. The mass resolution is set at 60,000 for MS and 30,000 for MS/MS scans. Dynamic exclusion is used to improve the duty cycle.

#### Data analysis

The raw data are extracted and searched using MaxQuant v1.6.17.0. MS data were searched against a UniProt *B. subtilis* (strain 168) proteome (4271 protein sequences). A 1:1 concatenated forward-reverse database was constructed to calculate the false discovery rate. Common contaminant protein sequences were included in the database. There are 8568 total protein sequences in the database. Search parameters are set to MaxQuant default settings with the enzyme parameter limited to full tryptic peptides with a maximum miscleavage of 1. Total TMT reporter intensities were used for relative protein quantitation. Isotope impurities of TMTpro reagents were corrected using correction factors provided by the manufacturer (Thermo Fisher Scientific). Median normalization was performed to normalize the protein TMT reporter intensities in which the log ratios between different TMTs are adjusted globally such that the median log ratio is zero. To determine relative levels of proteins, results were divided by the level for the WT samples, averaged between replicates, and expressed as fold changes.

### Ribosome sedimentation profiles

The absorbance of the clarified lysates at 260 nm was determined, and 40 absorbance units were loaded on to sucrose gradients. Sucrose gradients (10 to 40%) were made in the sucrose buffer [20 mM tris-HCl (pH 7.6), 15 mM magnesium acetate, 100 mM ammonium acetate, 0.1 mM dithiothreitol, 2 mM phenylmethylsulfonyl fluoride, 10 or 40% sucrose (w/v), and diethyl pyrocarbonate–treated water] using a Gradient Master (BioComp Instruments) and prechilled at 4°C. Following centrifugation at 19,500 rpm at 4°C for 17.5 hours (SW 41 Ti rotor, Beckman), the gradients were analyzed by measuring the absorbance at 260 nm using a Piston Gradient Fractionator (BioComp Instruments).

### ATP measurements

Steps described in the ATP Bioluminescence Assay Kit HS II (Roche Scientific, 11-699-709-001) protocol were followed for the sample and standard preparation. Specifically, 5 × 10^5^ cells were taken in a 50-μl volume to a 96-well plate. Fifty microliters of the lysis buffer was added to each sample for room temperature incubation for 5 min. One hundred microliters of the luciferase reagent was added to each sample, immediately followed by luminescence measurements using a Spark Multimode Microplate Reader (Tecan). A standard curve was generated for every experiment concurrently with samples. The luminescence intensities of samples were converted to concentration values using the standard curve. ATP concentration per cell was calculated with the assumption that an OD_600_ of 1.0 is 5 × 10^9^ cells/ml and that the volume of a cell is 0.9 fl.

## Supplementary Materials

### This PDF file includes:

Supplementary Text Figs. S1 to S7 Tables S1 to S4 References
