# Supplementary material for: Physiological cost of antibiotic resistance: Insights from a ribosome variant in bacteria
Source: Sci Adv. 2024 Nov 15;10(46):eadq5249. doi: 10.1126/sciadv.adq5249 (PMC11567004; doi:10.1126/sciadv.adq5249)
Supplement: Supplementary file 1 — Supplementary Text Figs. S1 to S7 Tables S1 to S4 References [file sciadv.adq5249_sm.pdf]

Science Advances
AAAS

Supplementary Materials for

Physiological cost of antibiotic resistance: Insights from a ribosome variant in bacteria

Eun Chae Moon *et al.*

Corresponding author: Gürol M. Süel, gsuel@ucsd.edu

Sci. Adv. **10**, eadq5249 (2024)
DOI: 10.1126/sciadv.adq5249

This PDF file includes:

Supplementary Text
Figs. S1 to S7
Tables S1 to S4
References

Supplementary Text

Dynamic Flexibility Index (DFI)

Dynamic Flexibility Index (DFI) (42–45, 65, 66) is a novel metric that quantifies the relative vibrational entropy of residues by calculating the resilience each residue experiences to perturbations in the molecule. Computation of DFI utilizes Perturbation Response Scanning (PRS) (67) technique using random perturbative forces as probes to sample the local vibrational ensemble of each residue. These perturbative forces emulate the effect of the stochastic nature of forces that exists in proteins due to their interaction with the thermal bath, solvent molecules, and other small molecules in a cell.

In order to calculate the DFI, the interactions between amino acids in a protein are coarse-grained using the Elastic Network Model (ENM). According to this model, the complexity of interactions among various pairs of residues (amino acids or nucleotides) is reduced to single point node-to-node interactions. In these, a node is represented by an alpha-Carbon (Cα) atom in the case of amino acid or a Phosphorus atom in the case of ribosomal nucleotides. Each node in this network is interacting with all the other nodes where the interaction is represented by a harmonic spring whose force constant inversely scales with the 6th power of the pairwise distance between them (42, 43). Therefore, the positions that are very far away from each other have very small interaction energy between them and vice versa. Furthermore, the interactions between the ribosomal assembly and the metallic ions (magnesium and zinc) crystalized with the ribosome are also included by representing them with another node and joining them with the rest of the nodes with the help of similar harmonic springs.

Using this model, the response of the ribosomal assembly in its native state to a perturbative force ($\boldsymbol{F}$) can be calculated using Linear Response Theory (LRT) (39) as:

$$\Delta \boldsymbol{R}_{3N\times 1} = \boldsymbol{H}^{-1}_{3N\times 3N} \boldsymbol{F}_{3N\times 1} \quad \text{[S1]}$$

where $\boldsymbol{H}^{-1}$ is the inverse of the $3N$x$3N$ Hessian matrix of the molecule with N interacting nodes. It is composed of the second-order derivatives of the harmonic potentials with respect to the components of the position vectors of the nodes, giving the position co-variance of the residue pairs in the static conformation. $\boldsymbol{\Delta R}$ is the response vector of residues due to the perturbative force. Here, we use random unit Brownian kicks as perturbative forces in order to sample the vibrational landscape of the molecule.

Most protein systems are composed of several hundred residues (each represented by a node), in which case calculating the inverse of hessian is a trivial and computationally inexpensive job for most recent computers. However, a typical ribosomal assembly is composed of a large number of small to fairly large proteins interacting with RNA molecules. In total, the number of residues including metal ions in the assembly is in the order of ten thousand (10380 for 4v56 ribosome (68)). This makes the Hessian matrix very large (31140 x 31140) and expensive to store in the memory (~40 GB if using float32). In addition, performing eigenvalue decomposition for inversion of such large matrices is not possible given the architecture of most high memory machines. Therefore, we used ARPACK (69) which is designed to compute a set of eigenvalues and corresponding eigenvectors numerically for a sparse matrix (which a Hessian is). It uses a

method called Implicitly Restarted Lanczos Method (70) to recursively estimate the largest 20 eigenvalues and corresponding eigenvectors within the lowest possible tolerance (based on machine precision). These are then used to estimate the inverse of the hessian matrix. This method was initially tested on some other proteins of smaller sizes ranging from 200-550 residues. It failed to produce any noticeable differences in the final DFI profiles.

Using the above-described methods, a large number of such random perturbations, uniformly distributed in space, are applied to each node and the response of these forces are averaged over in order to calculate an "isotropic" response profile of the node. This process is performed sequentially to each node in the coarse-grained ribosomal assembly in order to calculate the Perturbation Response Matrix, A as,

$$A_{N\times N} = \begin{bmatrix} |\Delta R^1|_1 & \cdots & |\Delta R^N|_1 \\ \vdots & \ddots & \vdots \\ |\Delta R^1|_N & \cdots & |\Delta R^N|_N \end{bmatrix} \quad \text{[S2]}$$

where $\left|\Delta R^j\right|_i = \sqrt{\langle(\Delta R)^2\rangle}$ is the magnitude of fluctuation response at site 'i' due to the perturbations at site 'j' averaged over random perturbations in all directions. Thereafter, the DFI score of a residue position 'i' is defined as the ratio of its net response as all the residues in the molecule are perturbed one by one in a sequential manner and the net displacement of all the residues when everything is perturbed.

$$DFI_i = \frac{\sum_{j=1}^{N}\left|\Delta R^j\right|_i}{\sum_{i=1}^{N}\sum_{j=1}^{N}\left|\Delta R^j\right|_i} \quad \text{[S3]}$$

Therefore, a residue with a higher DFI score is more susceptible to random perturbations in the protein and samples the local conformation space more freely, hence is labeled as a "flexible" residue. On the other hand, residues with a lower DFI score, are more resilient to motions in the protein and are therefore called "rigid" residues.

L22 protein is a globular protein that is embedded on the surface of the large ribosomal subunit. The L22 protein comprises a loop that penetrates the core of the ribosome. The insertion of 7 residues in the L22* mutation doubles the number of residues in this loop. We used AlphaFold (50, 51) to model L22* variant.

The deletion of L34 protein in the ribosome would create a cavity in the ribosomal assembly. Due to this cavity, the other amino acids surrounding and interacting with the L34 protein would now have fewer interacting partners. Thus, we focus on other regions in the ribosomal assembly with a cavity to model the interaction of the nodes (residues/nucleotides) exposed to the cavity. One such cavity can be found at the exit tunnel of the ribosome. We picked some residue positions on the surface of the cavity (residue positions 67-73 in protein L4 and 460-462, 470-472, 1257-1259 in ribosomal polymer 22) and analyzed the average distribution of their interactions with distance. Afterward, using it as a reference, we downscaled the strength of interactions of each residue position on the surface of the cavity created by the deletion of L34 protein. The original distribution of the strength of interactions of these surface residues (Fig. S5A, solid black line), the reference distribution (solid blue line), and the distribution post-scaling (broken black line) are shown for

nucleotides 126, 683, and 1309 in ribosomal polymer 23. It should be noted that since the cavity created by the deletion of L34 is relatively narrower, we only focus on scaling interactions within 6Å from the surface residues. This avoids including interactions from residues from the other side of the cavity.

<u>The coarse-grained computational ENM model captures the essence of all atomistic Molecular Dynamics simulation</u>

In order to test the validity of our method, we compared the root mean square fluctuations (RMSF) in the Cα and phosphorus atoms of the ribosome predicted by our coarse-grained ENM model with that observed in an all atomistic MD simulation. For this, we used the data from MD trajectories calculated in a study by Malte Warias, et. al. (71). In the study, the authors have used the cryo-EM structure of an *Escherichia coli* ribosome in complex with tRNAs, EF-Tu, and GTP as the starting structure for the MD simulation (protein data bank, PDB: 5uym (72)). The simulations borrowed the force field parameters from amber99sb force field (73) and the SPC/E water model (74). Two 2μs long independent simulations were run initialized from the same initial structure (please visit (71) for further details regarding the MD simulation).

Using the last 1μs of the two trajectories, we calculated the RMSF of each Cα and phosphorus atoms in the ribosome. These are then compared with the RMSF predicted by modeling the interactions in the structure from the same ribosome (PDB: 5uym) using the coarse-grained ENM network as described earlier. From this, we observe that through the coarse-grained ENM model we are able to capture the salient features of the RMSF profile from MD, thereby mimicking the dynamics efficiently (Fig. S4).

<u>Mg^{2+} pooling model</u>

We consider three potential states in which Mg^{2+} can reside within *B. subtilis*: free, bound to ribosomes, and bound to ATPs. The corresponding chemical reactions are:

$$\mathrm{Mg} + \mathrm{R_i} \underset{\mathrm{k_{rui}}}{\overset{\mathrm{k_{rbi}}}{\rightleftarrows}} \mathrm{R_{i+1}} \qquad [S4]$$

$$\mathrm{Mg} + ATP \underset{\mathrm{k_{au}}}{\overset{\mathrm{k_{ab}}}{\rightleftarrows}} \mathrm{Mg \cdot ATP} \qquad [S5]$$

Here reaction [S4] runs for i = 0, 1, …, N-1, with R_i representing the ribosome bound to i Mg^{2+}. Given the indistinguishable nature of the Mg^{2+} binding sites, the combinatorics of this reaction dictate that

$$k_{rbi} = (N - i)k_{rb}, \quad k_{rui} = (i+1)k_{ru} \qquad [S6]$$

where N is the maximum number of Mg^{2+} that the ribosome can hold, and we have defined k_{rb} and k_{ru} as basal binding and unbinding rates between ribosomes and Mg^{2+}. In what follows, we will use M to represent the concentration of free Mg^{2+} inside the cell, with a denoting the

concentration of available ATP, and A the concentration of ATP bound to Mg^{2+}. The following conservation laws must hold:

$$a + A = A_t \quad [S7]$$
$$\sum_{i=0}^{N} R_i = R_t \quad [S8]$$
$$M + A + \sum_{i=1}^{N} iR_i = M_t \quad [S9]$$

Our goal is to determine how the total Mg^{2+} in the cell is distributed among the three different pools listed above, which correspond to the three terms on the left-hand side of Eq. [S9]. In particular, we aim to determine how these three terms depend on the total Mg^{2+} concentration in the cell, and eventually on the extracellular Mg^{2+} concentration (which we can control experimentally).

We first note that the dynamics of the system can be described by means of the following set of coupled ordinary differential equations:

$$\frac{dA}{dt} = k_{ab}M(A_t - A) - k_{au}A \quad [S10]$$
$$\frac{dR_0}{dt} = -k_{rb0}MR_0 + k_{ru0}R_1 \quad [S11]$$
$$\frac{dR_i}{dt} = -k_{rbi}MR_i + k_{rui}R_{i+1} + k_{rb(i-1)}MR_{(i-1)} - k_{ru(i-1)}R_i, \quad i = 0,1,\ldots,N-1 \quad [S12]$$
$$\frac{dR_N}{dt} = k_{rb(N-1)}MR_{N-1} - k_{ru(N-1)}R_N \quad [S13]$$

<u>Mg^{2+} pooling model: steady-state solution</u>

Solving these equations in the steady-state leads to:

$$\bar{A} = \frac{A_t\bar{M}}{k_{ad} + \bar{M}} \quad [S14]$$
$$\bar{R}_{i+1} = \frac{\bar{M}\,\bar{R}_i}{k_{rdi}} \quad [S15]$$

where $k_{ad} \equiv k_{au}/k_{ab}$ while the expressions of k_{rbi} and k_{rui} given in Eq. [S6] lead to $k_{rdi} \equiv k_{rd}(i+1)/(N-i)$ with $k_{rd} \equiv k_{ru}/k_{rb}$. With this, Eq. [S15] transforms into:

$$R_{i+1} = \frac{M}{k_{rd}} R_i \frac{N-i}{i+1} \Longrightarrow R_i = \left(\frac{M}{k_{rd}}\right)^i R_0 \binom{N}{i} \quad [S16]$$

where we have dropped the bars that denote steady-state for simplicity. Introducing this expression for R_i into the conservation law [S8] and using the binomial theorem leads to the following expression for the concentration of naked ribosomes as a function of the total ribosome and free Mg^{2+} concentrations:

$$R_0 = \frac{R_t}{(1 + M/k_{rd})^N} \quad [S17]$$

Equations [S16] and [S17] allow us to determine the concentration of Mg^{2+} that is bound to ribosomes in the cell:

$$\sum_{i=0}^{N} iR_i = \frac{R_t}{(1 + M/k_{rd})^N} \sum_{i=1}^{N} i \left(\frac{M}{k_{rd}}\right)^i \binom{N}{i} \qquad [S18]$$

The sum in this expression can be computed through the moment-generating function of the binomial distribution, resulting in

$$\sum_{i=1}^{N} i \left(\frac{M}{k_{rd}}\right)^i \binom{N}{i} = N \frac{M}{k_{rd}} \left(1 + \frac{M}{k_{rd}}\right)^{N-1} \qquad [S19]$$

Introducing Eq. [S19] into expression [S18] leads to the final expression of the concentration of Mg^{2+} bound to ribosomes:

$$\sum_{i=0}^{N} iR_i = \frac{NR_t M}{k_{rd}+M} \qquad [S20]$$

Using Eqs. [S14] and [S20], the Mg^{2+} conservation law [S9] transforms into the final balance equation for the three Mg^{2+} pools in the cell:

$$M_t = M + \frac{A_t M}{k_{ad} + M} + \frac{NR_t M}{k_{rd} + M} \qquad [S21]$$

where the three terms are free Mg^{2+}, ATP-bound Mg^{2+}, and ribosome-bound Mg^{2+}, respectively (Fig. 4A).

Finally, we also assume that the total ATP and ribosome levels depend on the Mg^{2+} concentration, to account for a potential upregulation of MgtC homolog in response to Mg^{2+} deficiency (29):

$$A_t \to \frac{A_t M}{K_a + M}, \quad R_t \to \frac{R_t M}{K_r + M} \qquad [S22]$$

Our aim is to solve Eq. [S21] to determine how Mg^{2+} is distributed among the three different pools (free, ATP, and ribosome) as a function of extracellular concentration. First, we need to model how the total intracellular Mg^{2+} concentration M_t depends on the extracellular Mg^{2+} concentration M_E. We assume a hyperbolic dependence between the two quantities, corresponding to Michaelis-Menten-like kinetics that eventually saturates:

$$M_t = \frac{\alpha M_E}{K_m + M_E} \qquad [S23]$$

Using Eq. [S23] we can solve numerically Eq. [S21] to obtain the concentration of ATP-bound Mg^{2+} as a function of extracellular Mg^{2+} concentration, which we can then compare with the experimental observation, as shown in Fig. 4A and Fig. 4C. Results are plotted for both the WT and the L22* mutation. The effect of the mutation is to increase the affinity between Mg^{2+} and ribosomes, the concentration of ribosomes, and the efficiency of Mg^{2+} uptake (see parameters in Table S4).

The energy difference between the binding of Mg^{2+} with ATP and ribosome

At the steady-state conditions described in the model above the dissociation constant of Mg^{2+} binding with ATP (k_{ad}) and the dissociation constant of Mg^{2+} binding with the ribosome (k_{rdi}), where 'i' represents the number of Mg^{2+} bound to the ribosome. Therefore, for these two reactions occurring at a steady state in a cell, the free energy difference between the binding of another Mg^{2+} with the ribosome and ATP can be expressed as:

$$\Delta\Delta G_{R_i-ATP} == -kTln\frac{k_{ad}}{k_{rdi}} \quad [S24]$$

and using Eq. [S6]:

$$\Delta\Delta G_{R_i-ATP} == -kTln\left[\frac{k_{ad}(N-i)}{k_{rd}(i+1)}\right] \quad [S25]$$

Here, we have used the Arrhenius rate law to describe the relationship between the free energies and the respective rate constants. Using the values for the parameters used earlier (Table S4), see that there are only two parameters we can vary in the expression for the free energy difference (Eq. [S25]) – the dissociation constant for binding between ribosome and Mg^{2+} (k_{rd}) and the dissociation constant for binding between ATP and Mg^{2+} (k_{ad}). Moreover, the dissociation constant for binding between ATP and Mg^{2+}, k_{ad}, is independent of the ribosome, and mutations in the ribosome do not affect this. Therefore, this parameter is treated as a constant in our model. We explore the outcomes of varying k_{rd} from 0.1 μM to 10 μM on the free energy difference. We observe that as expected, as k_{rd} increases, the binding of Mg^{2+} to ribosome becomes less favorable as compared to its binding with ATP (Fig. S6B).

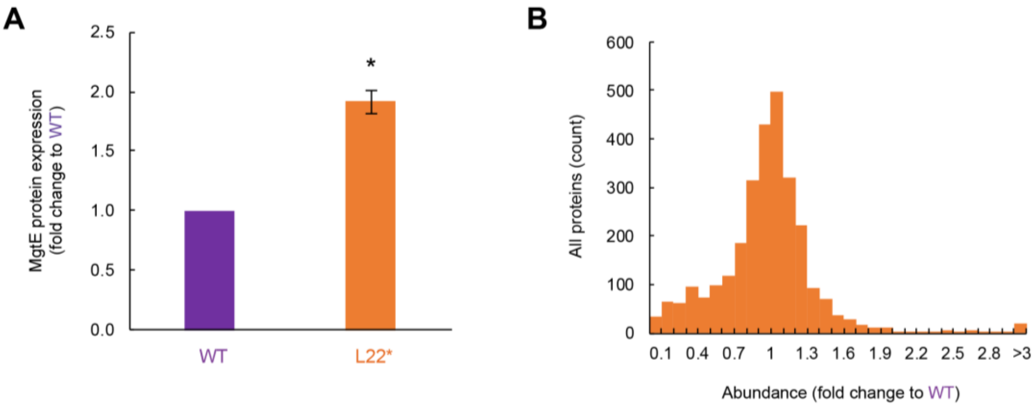

Fig. S1.

Proteomic analysis of WT and L22*, related to Fig. 2. (**A**) Relative fold change of MgtE protein in the L22* strain at 2 mM extracellular Mg^{2+} concentration relative to WT (mean ± 95% confidence interval; n = 2 samples for WT, n = 1 sample for L22*). The raw values are 7979079 and 7597869 for WT, and 14897466 for L22*. Unpaired t-test (t = 21.53, df = 1, P = 0.0295) shows statistical significance; *P < 0.05. (**B**) Histogram showing fold change of ribosomal protein levels in the L22* strain relative to WT at 2 mM extracellular Mg^{2+} concentration (n = 2 samples for WT, n = 1 sample for L22*). Using a repeated measures two-way ANOVA, main effects of strain (F[1,1] = 88.12, P = 0.0676) was not significant, and protein (F[2835,2835] = 1047, P < 0.0001) and interaction (F[2835,2835] = 54.27, P < 0.0001) were significant.

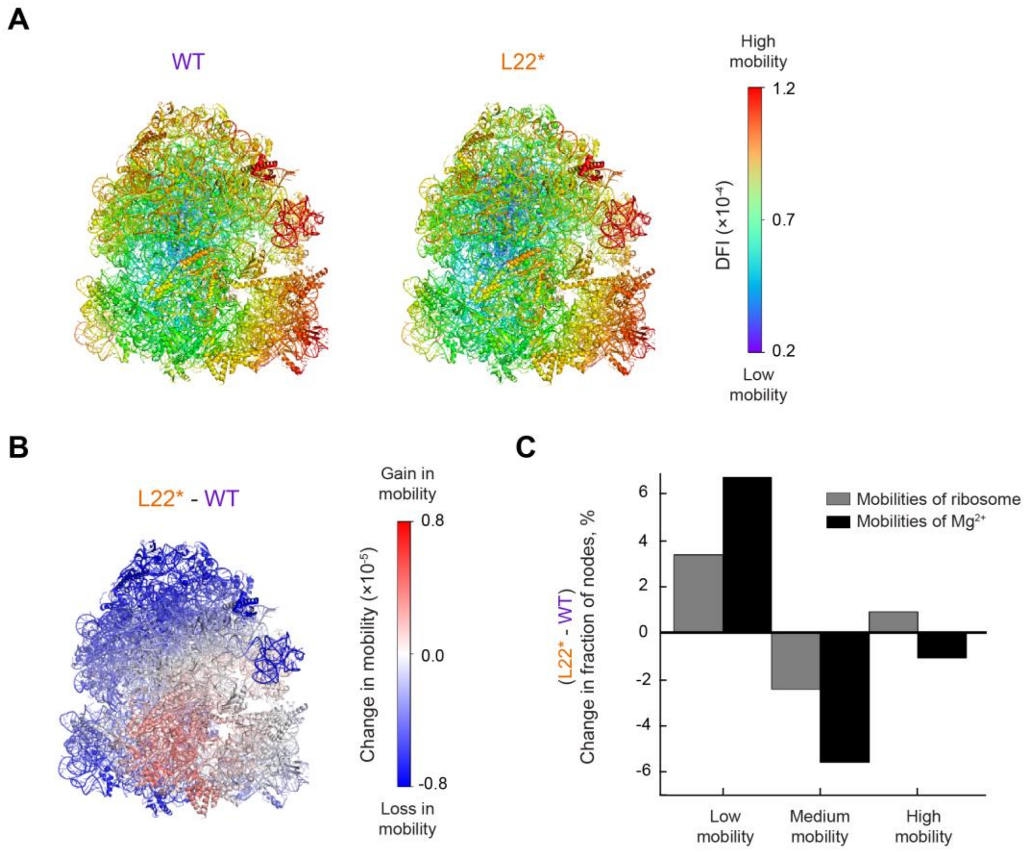

Fig. S2.

A dynamic flexibility index of ribosomal components reveals significant changes regarding association with Mg^{2+} in L22*, related to Fig. 3. (**A**) Cartoon illustrations of DFI of carbon and phosphorous (represent each amino acid and nucleotide, respectively) in WT and L22* ribosomes. A shared color scale among WT and L22* ribosome is at the right. The color scale is normalized to the maximum and minimum DFI values. (**B**) Ribosome cartoon of change in mobility as the differences of DFI of all nodes except Mg^{2+} in L22* ribosome from those in WT ribosome. (**C**) Change in the fraction of nodes for L22* ribosome from the low mobility, medium mobility, and high mobility groups of WT DFI. A higher fraction of Mg^{2+} either increase or decrease mobility compared to protein and rRNA components.

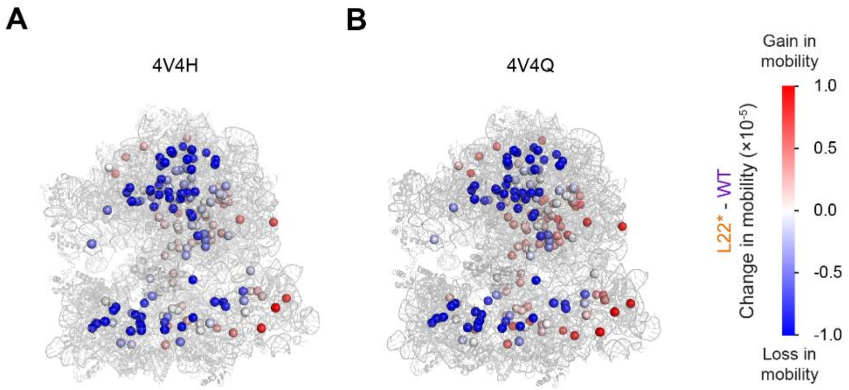

Fig. S3.

Change in the mobility of Mg^{2+} calculated from other bacterial ribosome structures mapped onto cartoon representation. A shared color bar is at the right. (**A**) 4V4H and (**B**) 4V4Q from *E. coli*.

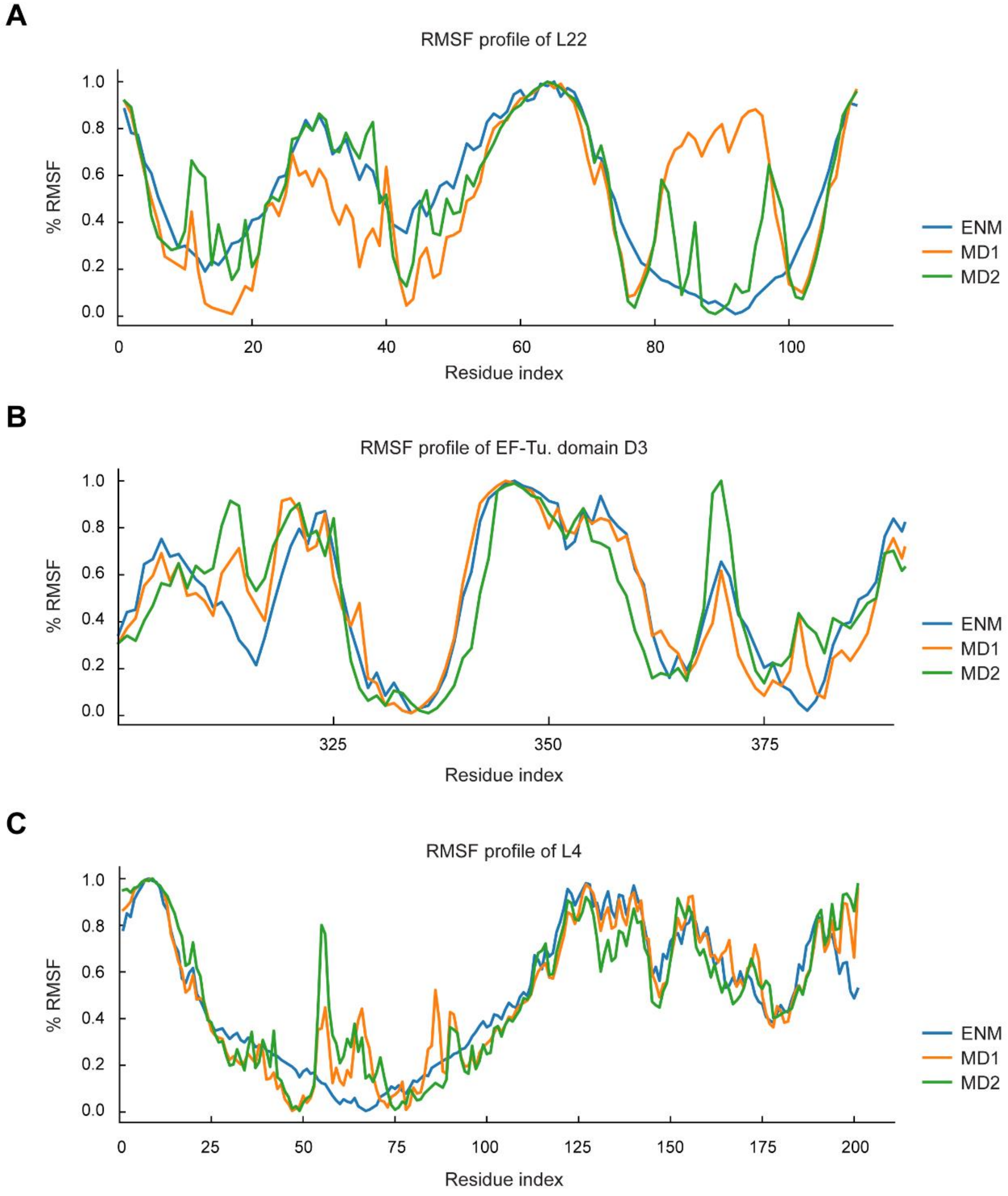

Fig. S4.

Comparing the root mean square fluctuations (RMSF) obtained from two independent all atomistic MD simulations of a ribosome (orange, MD1; green, MD2) with that predicted by a coarse-grained ENM model (blue). Here, as examples, we focus our comparison on three different regions of the ribosome, namely—(**A**) protein L22, (**B**) EF-Tu Domain D3, and (**C**) protein L4.

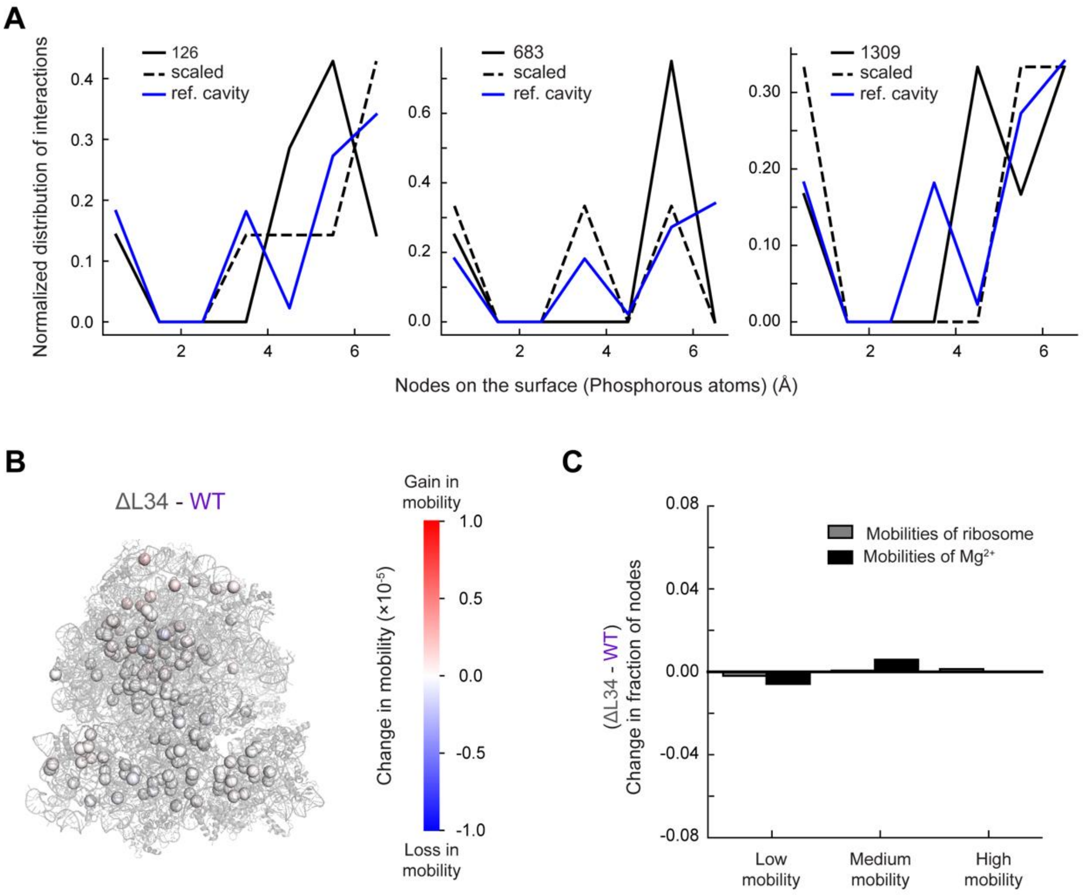

Fig. S5.

A dynamic flexibility index of ribosomal components reveals contrasting changes regarding association with Mg^{2+} in ΔL34, compared to L22*. (**A**) The radial distribution of contacts in L34 protein (black solid line) and that of another protein with a cavity (blue, solid). The interactions for these residues in ΔL34 are scaled up such that the radial distribution emulates that of the proteins in bulk (black, broken). (**B**) The ribosome cartoon on the left shows the DFI of Mg^{2+} in a ΔL34 ribosome. A color bar is to the right. (**C**) Change in the fraction of nodes for ΔL34 ribosome from the low mobility, medium mobility, and high mobility groups of WT DFI.

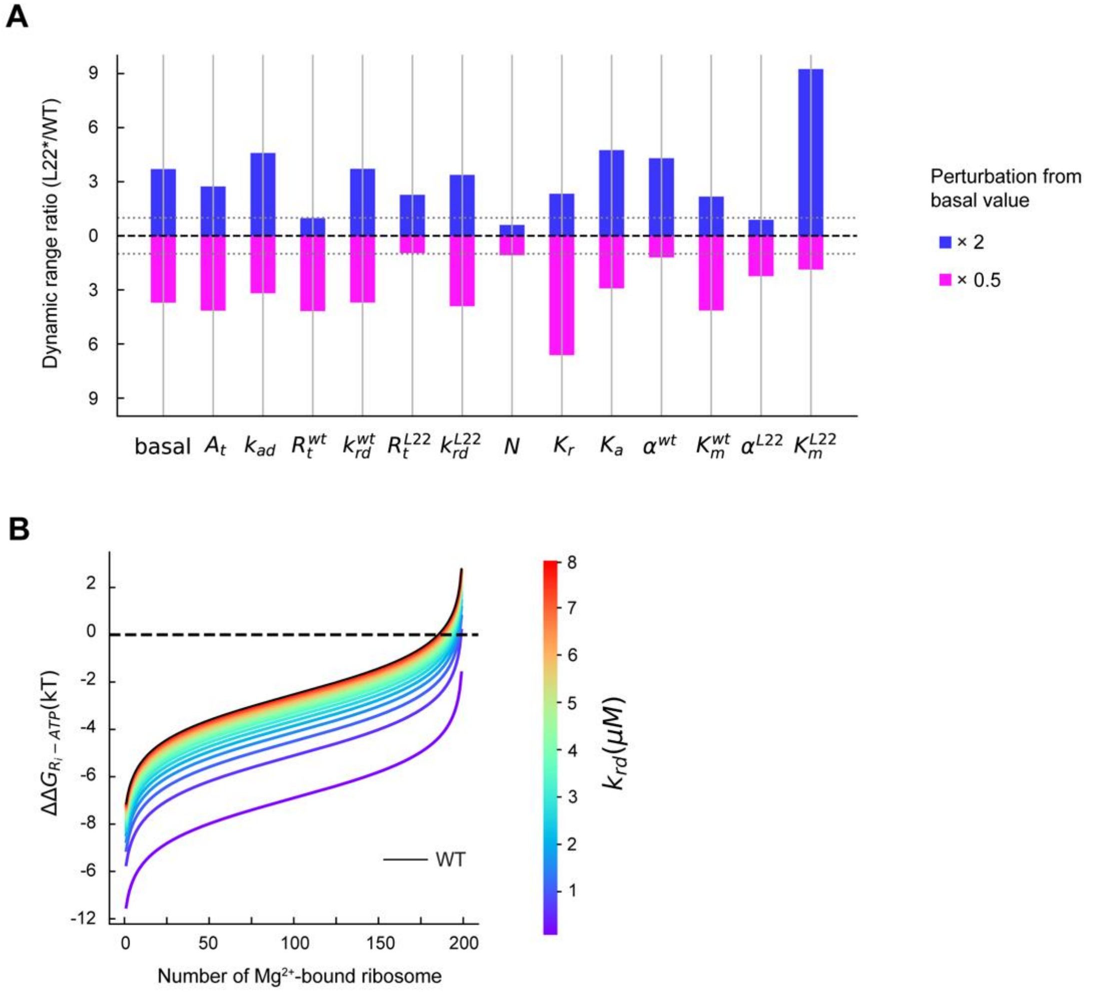

Fig. S6.

Mg^{2+} pooling model, related to Fig. 4A. (**A**) Dynamic range ratio between the L22 and WT strains for parameters in the Mg^{2+} pooling model. Dynamic range is defined here as the ratio between the maximum and minimum concentrations of ATP bound to Mg^{2+}, computed over the full range of extracellular Mg^{2+} values considered in Fig. 4A and 4C. The performance of the system is quantified by the ratio of the dynamics ranges exhibited by the L22* and WT strains. All parameters of the model have been perturbed by doubling and halving their basal values. The dynamic range ratio is larger than 1 (corresponding to the horizontal dotted gray line) for most parameter perturbations. (**B**) The WT ribosome (black) where k_{rd} is 8 μM. We then observe that as k_{rd} decreases, the binding of Mg^{2+} becomes more favorable.

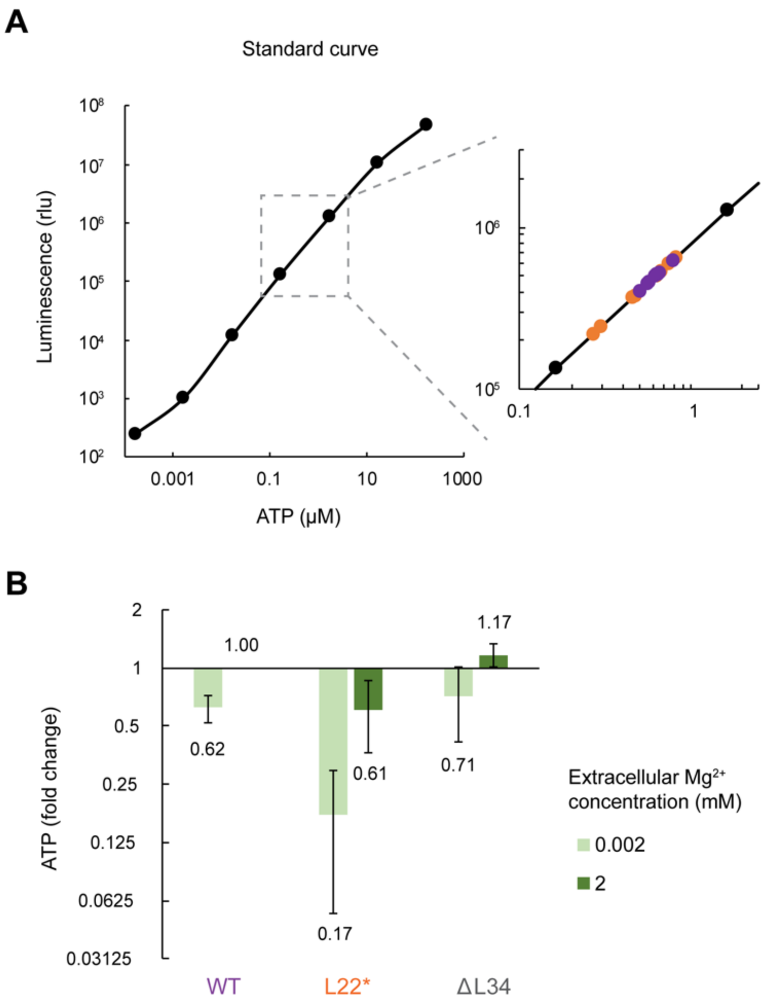

Fig. S7.

ATP measurements using bioluminescence, related to Fig. 4. (**A**) A standard curve for ATP measurements. Luminescence values of known ATP concentration generate a standard curve, which then fits with the data. (**B**) Fold change in ATP levels compared to WT 2mM extracellular Mg^{2+} condition (mean ± 95% confidence interval; n = 5 experiments for WT and L22*, n = 3 for ΔL34).

**Name**	**Source**	**BGSC ID**
Bacillus subtilis NCIB 3610 (WT)	Wade Winkler, University of Maryland	3A1
B. subtilis NCIB 3610 *rplV94* (L22*)	(8)	N/A
B. subtilis NCIB 3610 Δ*rpmH*::*cat* (ΔL34)	(19)	N/A
B. subtilis NCIB 3610 *sacA::*Mbox-*yfp*	This paper	N/A
B. subtilis NCIB 3610 *rplV94, sacA::*Mbox-*yfp*	This paper	N/A
B. subtilis NCIB 3610 *sacA::*Mbox^{M3}-*yfp*	This paper	N/A
B. subtilis NCIB 3610 *amyE::*P$_{hyperspank}$-*mKate2*	(75)	N/A
B. subtilis NCIB 3610 *rplV94* (L22*) *amyE::*P$_{hyperspank}$-*yfp*	This paper	N/A

Table S1.

Organisms and strains used in the study.

Name	Sequence	Source
GS450	ATGAGCAAAGGTGAAGAACTGTTCACC	This paper
GS351	GTCGCTACCATTACCAGTTGGTCTGG	This paper
GS2462	ccaactggtaatggtagcgacGTTTTGTTCCGTAATTGTGATGTAAGCGC	This paper
GS2463	aacagttcttcacctttgctCATCGGGACTCGTACCTCCTCTACG	This paper
GS2465	ATCGACATAAccaGATTTTTAATGCAGCTGG	This paper
GS2472	GCATTAAAAATCtggTTATGTCGATGATTTCTGTTGACCCATTGGCGTCT	This paper

Table S2.
Primers used to generate vectors for reporter strains.

Accession	Protein	Gene	WT	WT	L22*
P21464	30S ribosomal protein S2	rpsB	4874663667	4663399977	5155449658
P42919	50S ribosomal protein L2	rplB	5117868901	5023005304	5742897414
Q06797	50S ribosomal protein L1	rplA	4619269519	4653596814	5778144820
P42920	50S ribosomal protein L3	rplC	4003349810	3901954901	4915521732
P12877	50S ribosomal protein L5	rplE	4170547315	4073910233	5690583779
P21465	30S ribosomal protein S3	rpsC	4829105110	4835260386	5270292916
P21467	30S ribosomal protein S5	rpsE	2598619984	2451003427	3014361198
P21469	30S ribosomal protein S7	rpsG	5005889546	4721855168	6220738057
P21466	30S ribosomal protein S4	rpsD	3504586183	3315503179	3790956791
P46898	50S ribosomal protein L6	rplF	3747362171	3799007804	4822613949
P42923	50S ribosomal protein L10	rplJ	3421137532	3196075691	3938806121
Q06796	50S ribosomal protein L11	rplK	2683219855	2730425589	3663615933
P37437	50S ribosomal protein L9	rplI	762673010	819367248	1221593689
P70974	50S ribosomal protein L13	rplM	3039781456	2907965014	4050593966
P20282	30S ribosomal protein S13	rpsM	1586759318	1620022478	2127902866
P21468	30S ribosomal protein S6	rpsF	1510538869	1619362871	2215793637
P20277	50S ribosomal protein L17	rplQ	2257728470	2432859598	3563580810
P21471	30S ribosomal protein S10	rpsJ	1737365555	1727275841	2317642055
P55873	50S ribosomal protein L20	rplT	3596572421	3724849162	4540746897
P0CI78	50S ribosomal protein L24	rplX	1315561746	1307176068	3218259815
P12879	30S ribosomal protein S8	rpsH	2259722174	2194745917	3302960564
P12875	50S ribosomal protein L14	rplN	2789187150	2815535323	3803747785
P19946	50S ribosomal protein L15	rplO	1613312695	1638362930	2023824238
P21472	30S ribosomal protein S12	rpsL	1640149996	1588050814	1701722124
P42921	50S ribosomal protein L4	rplD	2026300038	1920763157	2564983694
P02394	50S ribosomal protein L7/L12	rplL	9294112850	8688173953	10124362047
P42924	50S ribosomal protein L23	rplW	907088696	879161871	2781846362
P21474	30S ribosomal protein S16	rpsP	1467074909	1358823982	4584542649
P14577	50S ribosomal protein L16	rplP	659520000	696903555	817428952
P46899	50S ribosomal protein L18	rplR	2305846088	2434665349	3835464506
O31742	50S ribosomal protein L19	rplS	1446223526	1396043918	1599200888
P04969	30S ribosomal protein S11	rpsK	1059520917	1014191209	1265819085
P21476	30S ribosomal protein S19	rpsS	450314840	432162477	1237962330
P05657	50S ribosomal protein L27	rpmA	474065689	378761545	1779526386
P21475	30S ribosomal protein S18	rpsR	2819385903	3046776271	3381398568
P21470	30S ribosomal protein S9	rpsI	879916310	907157370	1175947063
P12874	30S ribosomal protein S17	rpsQ	681620390	640115940	2367565110
P42060	50S ribosomal protein L22	rplV	675296690	707740653	1168399663
P12873	50S ribosomal protein L29	rpmC	422414616	390922291	1218338968

P19947	50S ribosomal protein L30	rpmD	156733419	138905703	609392785
O34967	50S ribosomal protein L31 type B	rpmE2	144409814	170923103	392457949
P26908	50S ribosomal protein L21	rplU	1111211552	1181466211	1496194507
Q03223	50S ribosomal protein L31	rpmE	72037319	64782627	161468180
P21478	30S ribosomal protein S21	rpsU	180667778	138469610	819992808
P37807	50S ribosomal protein L28	rpmB	170342529	113776237	464410923
O31587	Alternate 30S ribosomal protein S14	rpsN2	22592877	20703576	62543454
O34687	50S ribosomal protein L32	rpmF	237538564	187334272	847366005
P21477	30S ribosomal protein S20	rpsT	341648105	277796829	1000170432
P21473	30S ribosomal protein S15	rpsO	2270112485	2307974852	2485023431
P56849	50S ribosomal protein L33 1	rpmGA	2527768	2389457	7138414
P12878	30S ribosomal protein S14	rpsN1	68340620	52193862	233858698
P55874	50S ribosomal protein L35	rpmI	243132639	128529009	709565748
Q06798	50S ribosomal protein L33 2	rpmGB	10706543	8834325	33924880
P20278	50S ribosomal protein L36	rpmJ	75462823	67680197	210413047
P05647	50S ribosomal protein L34	rpmH	6714268	4420487	21460876

Table S3.
Raw proteomics data of ribosomal proteins in WT and L22* strains at 2 mM extracellular Mg^{2+} concentration.

Parameter	Description	Value	Reference
A_t	Maximum ATP concentration	2.5 mM	(55)
R_t	Maximum ribosome concentration	40 μM (WT)<br>90 μM (L22*)	(30, 55)
k_{ad}	Mg^{2+}-ATP dissociation constant	0.1 mM	(30)
k_{rd}	Mg^{2+}-ribosome dissociation constant	8 μM (WT)<br>5 μM (L22*)	
N	Maximum number of Mg^{2+} per ribosome	200	(16)
a	Intracellular Mg^{2+} concentration at saturation	18 mM (WT)<br>20 mM (L22*)	(76)
K_m	Michaelis constant of Mg^{2+} import	1 μM (WT)<br>0.9 μM (L22*)	
K_r	Coefficient of ribosome dependence on Mg^{2+}	0.05 mM	
K_a	Coefficient of ATP dependence on Mg^{2+}	0.5 mM	

Table S4.

Parameter values of the Mg^{2+} pooling model.

REFERENCES AND NOTES

1. J. Poehlsgaard, S. Douthwaite, The bacterial ribosome as a target for antibiotics. *Nat. Rev. Microbiol.* **3**, 870–881 (2005).

2. R. A. Sharrock, T. Leighton, H. G. Wittmann, Macrolide and aminoglycoside antibiotic resistance mutations in the Bacillus subtilis ribosome resulting in temperature-sensitive sporulation. *Mol. Gen. Genet.* **183**, 538–543 (1981).

3. S. H. Thorbjarnardóttir, R. Á. Magnúsdóttir, G. Eggertsson, S. A. Kagan, Ó. S. Andrésson, Mutations determining generalized resistance to aminoglycoside antibiotics in *Escherichia coli*. *Mol. Gen. Genet.* **161**, 89–98 (1978).

4. P. Buckel, A. Buchberger, A. Böck, H. G. Wittmann, Alteration of ribosomal protein L6 in mutants of *Escherichia coli* resistant to gentamicin. *Mol. Gen. Genet.* **158**, 47–54 (1977).

5. R. Nessar, J. M. Reyrat, A. Murray, B. Gicquel, Genetic analysis of new 16S rRNA mutations conferring aminoglycoside resistance in *Mycobacterium abscessus*. *J. Antimicrob. Chemother.* **66**, 1719–1724 (2011).

6. D. Criswell, V. L. Tobiason, J. S. Lodmell, D. S. Samuels, Mutations conferring aminoglycoside and spectinomycin resistance in *Borrelia burgdorferi*. *Antimicrob. Agents Chemother.* **50**, 445–452 (2006).

7. S. Chiba, A. Lamsa, K. Pogliano, A ribosome-nascent chain sensor of membrane protein biogenesis in *Bacillus subtilis*. *EMBO J.* **28**, 3461–3475 (2009).

8. D. D. Lee, L. Galera-Laporta, M. Bialecka-Fornal, E. C. Moon, Z. Shen, S. P. Briggs, J. Garcia-Ojalvo, G. M. Süel, Magnesium flux modulates ribosomes to increase bacterial survival. *Cell* **177**, 352–360.e13. (2019).

9. P. Sander, B. Springer, T. Prammananan, A. Sturmfels, M. Kappler, M. Pletschette, E. C. Böttger, Fitness cost of chromosomal drug resistance-conferring mutations. *Antimicrob. Agents Chemother.* **46**, 1204–1211 (2002).

10. A. H. Melnyk, A. Wong, R. Kassen, The fitness costs of antibiotic resistance mutations. *Evol. Appl.* **8**, 273–283 (2015).

11. D. I. Andersson, D. Hughes, Antibiotic resistance and its cost: Is it possible to reverse resistance? *Nat. Rev. Microbiol.* **8**, 260–271 (2010).

12. S. Hernando-Amado, F. Sanz-García, P. Blanco, J. L. Martínez, Fitness costs associated with the acquisition of antibiotic resistance. *Essays Biochem.* **61**, 37–48 (2017).

13. D. Drygin, R. A. Zimmermann, Magnesium ions mediate contacts between phosphoryl oxygens at positions 2122 and 2176 of the 23S rRNA and ribosomal protein L1. *RNA* **6**, 1714–1726 (2000).

14. D. J. Klein, P. B. Moore, T. A. Steitz, The contribution of metal ions to the structural stability of the large ribosomal subunit. *RNA* **10**, 1366–1379 (2004).

15. A. S. Petrov, C. R. Bernier, C. Hsiao, C. D. Okafor, E. Tannenbaum, J. Stern, E. Gaucher, D. Schneider, N. V. Hud, S. C. Harvey, L. D. Williams, RNA-magnesium-protein interactions in large ribosomal subunit. *J. Phys. Chem. B* **116**, 8113–8120 (2012).

16. B. S. Schuwirth, M. A. Borovinskaya, C. W. Hau, W. Zhang, A. Vila-Sanjurjo, J. M. Holton, J. H. D. Cate, Structures of the bacterial ribosome at 3.5 Å resolution. *Science* **310**, 827–834 (2005).

17. T. Zou, S. B. Ozkan, S. Cavagnero, Electrostatic effect of the ribosomal surface on nascent polypeptide dynamics. *ACS Chem. Biol.* **8**, 1195–1204 (2013).

18. Z. Guo, M. Gibson, S. Sitha, S. Chu, U. Mohanty, Role of large thermal fluctuations and magnesium ions in t-RNA selectivity of the ribosome. *Proc. Natl. Acad. Sci. U.S.A.* **108**, 3947–3951 (2011).

19. A. Rozov, I. Khusainov, K. E. Omari, R. Duman, V. Mykhaylyk, M. Yusupov, E. Westhof, A. Wagner, G. Yusupova, Importance of potassium ions for ribosome structure and function revealed by long-wavelength X-ray diffraction. *Nat. Commun.* **10**, 2519 (2019).

20. K. M. Fagerbakke, S. Norland, M. Heldal, The inorganic ion content of native aquatic bacteria. *Can. J. Microbiol.* **45**, 304–311 (1999).

21. R. L. Weiss, B. W. Kimes, D. R. Morris, Cations and ribosome structure. III. Effects on the 30S and 50S subunits of replacing bound Mg^{2+} by inorganic cations. *Biochemistry* **12**, 450–456 (1973).

22. K. H. Nierhaus, Mg^{2+}, K^{+}, and the ribosome. *J. Bacteriol.* **196**, 3817–3819 (2014).

23. B. J. McCarthy, The effects of magnesium starvation on the ribosome content of *Escherichia coli*. *Biochim. Biophys. Acta* **55**, 880–889 (1962).

24. R. F. Gesteland, Unfolding of *Escherichia coli* ribosomes by removal of magnesium. *J. Mol. Biol.* **18**, 356–371 (1966).

25. G. Blaha, N. Burkhardt, K. H. Nierhaus, Formation of 70S ribosomes: Large activation energy is required for the adaptation of exclusively the small ribosomal subunit. *Biophys. Chem.* **96**, 153–161 (2002).

26. A. Liiv, M. O'Connor, Mutations in the intersubunit bridge regions of 23 S rRNA. *J. Biol. Chem.* **281**, 29850–29862 (2006).

27. F. I. Wolf, A. Cittadini, Chemistry and biochemistry of magnesium. *Mol. Aspects Med.* **24**, 3–9 (2003).

28. M. Montero, G. Eydallin, A. M. Viale, G. Almagro, F. J. Muñoz, M. Rahimpour, M. T. Sesma, E. Baroja-Fernández, J. Pozueta-Romero, *Escherichia coli* glycogen metabolism is controlled by the PhoP-PhoQ regulatory system at submillimolar environmental Mg^{2+} concentrations, and is highly interconnected with a wide variety of cellular processes. *Biochem. J.* **424**, 129–141 (2009).

29. M. H. Pontes, J. Yeom, E. A. Groisman, Reducing ribosome biosynthesis promotes translation during low Mg^{2+} stress. *Mol. Cell* **64**, 480–492 (2016).

30. M. H. Pontes, A. Sevostyanova, E. A. Groisman, When too much ATP is bad for protein synthesis. *J. Mol. Biol.* **427**, 2586–2594 (2015).

31. N. Nagai, T. Fukuhata, Y. Ito, Effect of magnesium deficiency on intracellular ATP levels in human lens epithelial cells. *Biol. Pharm. Bull.* **30**, 6–10 (2007).

32. Y. Shindo, R. Yamanaka, K. Suzuki, K. Hotta, K. Oka, Intracellular magnesium level determines cell viability in the MPP^+ model of Parkinson's disease. *Biochim. Biophys. Acta* **1853**, 3182–3191 (2015).

33. C. E. Dann, C. A. Wakeman, C. L. Sieling, S. C. Baker, I. Irnov, W. C. Winkler, Structure and mechanism of a metal-sensing regulatory RNA. *Cell* **130**, 878–892 (2007).

34. Z. Kurkcuoglu, I. Bahar, P. Doruker, ClustENM: ENM-based sampling of essential conformational space at full atomic resolution. *J. Chem. Theory Comput.* **12**, 4549–4562 (2016).

35. K. Chang, J. Wen, L. Yang, Functional importance of mobile ribosomal proteins. *Biomed. Res. Int.* **2015**, 1–11 (2015).

36. Y. Wang, A. J. Rader, I. Bahar, R. L. Jernigan, Global ribosome motions revealed with elastic network model. *J. Struct. Biol.* **147**, 302–314 (2004).

37. M. T. Zimmermann, K. Jia, R. L. Jernigan, Ribosome mechanics informs about mechanism. *J. Mol. Biol.* **428**, 802–810 (2016).

38. F. Tama, M. Valle, J. Frank, C. L. Brooks III, Dynamic reorganization of the functionally active ribosome explored by normal mode analysis and cryo-electron microscopy. *Proc. Natl. Acad. Sci. U.S.A.* **100**, 9319–9323 (2003).

39. M. Ikeguchi, J. Ueno, M. Sato, A. Kidera, Protein structural change upon ligand binding: Linear response theory. *Phys. Rev. Lett.* **94**, 078102 (2005).

40. M. M. Tirion, Large amplitude elastic motions in proteins from a single-parameter, atomic analysis. *Phys. Rev. Lett.* **77**, 1905–1908 (1996).

41. I. Bahar, T. R. Lezon, L. Yang, E. Eyal, Global dynamics of proteins: Bridging between structure and function. *Annu. Rev. Biophys.* **39**, 23–42 (2010).

42. Z. N. Gerek, S. Kumar, S. Banu Ozkan, Structural dynamics flexibility informs function and evolution at a proteome scale. *Evol. Appl.* **6**, 423–433 (2013).

43. Z. N. Gerek, O. Keskin, S. B. Ozkan, Identification of specificity and promiscuity of PDZ domain interactions through their dynamic behavior. *Proteins* **77**, 796–811 (2009).

44. P. Campitelli, T. Modi, S. Kumar, S. B. Ozkan, The role of conformational dynamics and allostery in modulating protein evolution. *Annu. Rev. Biophys.* **49**, 267–288 (2020).

45. T. Modi, J. Huihui, K. Ghosh, S. B. Ozkan, Ancient thioredoxins evolved to modern-day stability-function requirement by altering native state ensemble. *Philos. Trans. R. Soc. Lond. B Biol. Sci.* **373**, 20170184 (2018).

46. P. Campitelli, L. Swint-Kruse, S. B. Ozkan, Substitutions at nonconserved rheostat positions modulate function by rewiring long-range dynamic interactions. *Mol. Biol. Evol.* **38**, 201–214 (2021).

47. I. C. Kazan, P. Sharma, M. I. Rahman, A. Bobkov, R. Fromme, G. Ghirlanda, S. B. Ozkan, Design of novel cyanovirin-N variants by modulation of binding dynamics through distal mutations. *elife* **11**, e67474 (2022).

48. J. Lu, M. I. Rahman, I. C. Kazan, N. R. Halloran, A. A. Bobkov, S. B. Ozkan, G. Ghirlanda, Engineering gain-of-function mutants of a WW domain by dynamics and structural analysis. *Protein Sci.* **32**, e4759 (2023).

49. N. J. Ose, P. Campitelli, T. Modi, I. C. Kazan, S. Kumar, S. B. Ozkan, Some mechanistic underpinnings of molecular adaptations of SARS-COV-2 spike protein by integrating candidate adaptive polymorphisms with protein dynamics. *elife* **12**, RP92063 (2023).

50. J. Jumper, R. Evans, A. Pritzel, T. Green, M. Figurnov, O. Ronneberger, K. Tunyasuvunakool, R. Bates, A. Žídek, A. Potapenko, A. Bridgland, C. Meyer, S. A. A. Kohl, A. J. Ballard, A.

Cowie, B. Romera-Paredes, S. Nikolov, R. Jain, J. Adler, T. Back, S. Petersen, D. Reiman, E. Clancy, M. Zielinski, M. Steinegger, M. Pacholska, T. Berghammer, S. Bodenstein, D. Silver, O. Vinyals, A. W. Senior, K. Kavukcuoglu, P. Kohli, D. Hassabis, Highly accurate protein structure prediction with AlphaFold. *Nature* **596**, 583–589 (2021).

51. M. Varadi, S. Anyango, M. Deshpande, S. Nair, C. Natassia, G. Yordanova, D. Yuan, O. Stroe, G. Wood, A. Laydon, A. Žídek, T. Green, K. Tunyasuvunakool, S. Petersen, J. Jumper, E. Clancy, R. Green, A. Vora, M. Lutfi, M. Figurnov, A. Cowie, N. Hobbs, P. Kohli, G. Kleywegt, E. Birney, D. Hassabis, S. Velankar, AlphaFold Protein Structure Database: Massively expanding the structural coverage of protein-sequence space with high-accuracy models. *Nucleic Acids Res.* **50**, D439–D444 (2022).

52. G. Akanuma, K. Yamazaki, Y. Yagishi, Y. Iizuka, M. Ishizuka, F. Kawamura, Y. Kato-Yamada, Magnesium suppresses defects in the formation of 70S ribosomes as well as in sporulation caused by lack of several individual ribosomal proteins. *J. Bacteriol.* **200**, e0021218 (2018).

53. C. G. England, E. B. Ehlerding, W. Cai, NanoLuc: A small luciferase is brightening up the field of bioluminescence. *Bioconjug. Chem.* **27**, 1175–1187 (2016).

54. B. He, J. D. Helmann, Metalation of extracytoplasmic proteins and bacterial cell envelope homeostasis. *Annu. Rev. Microbiol.* **78**, 10.1146/annurev-micro-041522-091507 (2024).

55. U. Moran, R. Phillips, R. Milo, SnapShot: Key numbers in biology. *Cell* **141**, 1262–1262.e1 (2010).

56. R. Mempin, H. Tran, C. Chen, H. Gong, K. K. Ho, S. Lu, Release of extracellular ATP by bacteria during growth. *BMC Microbiol.* **13**, 301 (2013).

57. D. A. Schneider, R. L. Gourse, Relationship between growth rate and ATP concentration in *Escherichia coli*: A bioassay for available cellular ATP. *J. Biol. Chem.* **279**, 8262–8268 (2004).

58. M. Scott, S. Klumpp, E. M. Mateescu, T. Hwa, Emergence of robust growth laws from optimal regulation of ribosome synthesis. *Mol. Syst. Biol.* **10**, 747 (2014).

59. E. Bosdriesz, D. Molenaar, B. Teusink, F. J. Bruggeman, How fast-growing bacteria robustly tune their ribosome concentration to approximate growth-rate maximization. *FEBS J.* **282**, 2029–2044 (2015).

60. J. L. Ross, The dark matter of biology. *Biophys. J.* **111**, 909–916 (2016).

61. H. Pi, B. M. Wendel, J. D. Helmann, Dysregulation of magnesium transport protects *Bacillus subtilis* against manganese and cobalt intoxication. *J. Bacteriol.* **202**, e00711–e00719 (2020).

62. A. W. Foster, T. R. Young, P. T. Chivers, N. J. Robinson, Protein metalation in biology. *Curr. Opin. Chem. Biol.* **66**, 102095 (2022).

63. W. Jahnen-Dechent, M. Ketteler, Magnesium basics. *Clin. Kidney J.* **5**, i3–i14 (2012).

64. I. Arganda-Carreras, V. Kaynig, C. Rueden, K. W. Eliceiri, J. Schindelin, A. Cardona, H. S. Seung, Trainable weka segmentation: A machine learning tool for microscopy pixel classification. *Bioinformatics* **33**, 2424–2426 (2017).

65. T. Zou, V. A. Risso, J. A. Gavira, J. M. Sanchez-Ruiz, S. B. Ozkan, Evolution of conformational dynamics determines the conversion of a promiscuous generalist into a specialist enzyme. *Mol. Biol. Evol.* **32**, 132–143 (2015).

66. T. Modi, S. B. Ozkan, Mutations utilize dynamic allostery to confer resistance in TEM-1 β-lactamase. *Int. J. Mol. Sci.* **19**, 3808 (2018).

67. C. Atilgan, A. R. Atilgan, Perturbation-response scanning reveals ligand entry-exit mechanisms of ferric binding protein. *PLoS Comput. Biol.* **5**, e1000544 (2009).

68. M. A. Borovinskaya, S. Shoji, J. M. Holton, K. Fredrick, J. H. D. Cate, A steric block in translation caused by the antibiotic spectinomycin. *ACS Chem. Biol.* **2**, 545–552 (2007).

69. R. B. Lehoucq, D. C. Sorensen, C. Yang, *ARPACK Users' Guide: Solution of Large-Scale Eigenvalue Problems with Implicitly Restarted Arnoldi Methods* (SIAM, 1998).

70. D. Calvetti, L. Reichel, A. Sorensen, An implicitly restarted Lanczos method for large symmetric eigenvalue problems. *Electron. Trans. Numer. Anal.* **2**, 1–21 (1994).

71. M. Warias, H. Grubmüller, L. V. Bock, tRNA dissociation from EF-Tu after GTP hydrolysis: Primary steps and antibiotic inhibition. *Biophys. J.* **118**, 151–161 (2020).

72. A. B. Loveland, G. Demo, N. Grigorieff, A. A. Korostelev, Ensemble cryo-EM elucidates the mechanism of translation fidelity. *Nature* **546**, 113–117 (2017).

73. V. Hornak, R. Abel, A. Okur, B. Strockbine, A. Roitberg, C. Simmerling, Comparison of multiple Amber force fields and development of improved protein backbone parameters. *Proteins* **65**, 712–725 (2006).

74. P. H. Poole, F. Sciortino, U. Essmann, H. E. Stanley, Phase behaviour of metastable water. *Nature* **360**, 324–328 (1992).

75. J. Humphries, L. Xiong, J. Liu, A. Prindle, F. Yuan, H. A. Arjes, L. Tsimring, G. M. Süel, Species-independent attraction to biofilms through electrical signaling. *Cell* **168**, 200–209.e12 (2017).

76. F.-X. Theillet, A. Binolfi, T. Frembgen-Kesner, K. Hingorani, M. Sarkar, C. Kyne, C. Li, P. B. Crowley, L. Gierasch, G. J. Pielak, A. H. Elcock, A. Gershenson, P. Selenko, Physicochemical properties of cells and their effects on intrinsically disordered proteins (IDPs). *Chem. Rev.* **114**, 6661–6714 (2014).
